# Wheat *VPE* Family: Identification, Evolution and Expression Associated with Grain Colour

**DOI:** 10.3390/biology15201800

**Published:** 2026-10-09

**Authors:** Yanzhen Wang, Yanqi Wang, Jiangtao Zhao, Jun Zheng, Menglin Lei, Ganggang Guo, Xia Liu

**Affiliations:** 1 Center for Agricultural Genetic Resources Research, Shanxi Agricultural University, Taiyuan 030031, China; wangyanzhen9605@163.com (Y.W.);; 2 College of Agriculture, Shanxi Agricultural University (Institute of Crop Sciences), Taigu 030801, China; 3 Seed Industry Development Center of Shanxi Province, Taiyuan 030006, China; 4 Zuoquan Modern Agricultural Industry Development Service Center, Zuoquan 032600, China; 5 State Key Laboratory of Crop Gene Resources and Breeding, Institute of Crop Sciences, Chinese Academy of Agricultural Sciences, Beijing 100081, China

**Keywords:** *Triticum aestivum*, vacuolar processing enzyme, whole-genome duplication, synteny, grain colour

## Abstract

Vacuolar processing enzymes (VPEs) are legumain-type cysteine proteases regulating plant protein processing, programmed cell death, seed maturation and stress responses. Nevertheless, the *VPE* family remains poorly characterized in hexaploid wheat (*Triticum aestivum* L.), and its possible roles in grain pigmentation are unclear. Here, we genome-wide identified 27 *TaVPE* genes and performed multi-omics analyses. Phylogenetic classification separated TaVPE proteins into four clades, all possessing conserved Peptidase_C13 and legumain_C domains, with similar motif patterns within each clade. These 27 *TaVPE* genes were unevenly located across 12 chromosomes of homoeologous groups 2, 3, 5 and 6. Collinearity analysis uncovered 26 intra-wheat collinear blocks and 47 orthologous gene pairs between wheat and rice, maize and foxtail millet, while no collinear counterparts were found in *Arabidopsis.* All duplicated pairs had Ka/Ks values less than 1, suggesting strong purifying selection. Numerous *cis*-acting elements related to hormones (ABA, MeJA, SA), stresses, and MYB/bHLH transcription factor binding sites were detected in *TaVPE* promoters. Expression data from public databases and transcriptomes of purple-grained Donghei-1 and white-grained Jinmai-66 (17, 31, 36 and 41 days after flowering) demonstrated predominant *TaVPE* expression at anthesis and grain-filling stages. Five *TaVPE* genes were retained as candidate genes associated with purple grain colour: *TaVPE6*, *TaVPE15*, *TaVPE18*, *TaVPE25* and *TaVPE26*. All five showed significantly higher expression in the purple-grained cultivar than in the white-grained one at one or more mid-to-late grain-filling stages. This work establishes the first systematic profile of wheat *VPE* family and identifies candidate genes potentially associated with anthocyanin accumulation, supporting future functional research and molecular breeding for anthocyanin-enriched wheat.

## Simple Summary

Purple wheat grains contain health-promoting anthocyanins, but the mechanisms controlling grain-coat anthocyanin accumulation remain unclear. Vacuolar processing enzymes (VPEs) are vacuolar proteases involved in grain-filling-related protein maturation and programmed cell death. We identified 27 *TaVPE* genes in bread wheat, assigned them to four groups and mapped them to 12 chromosomes. Transcriptome comparisons between purple- and white-grained wheat, verified by RT-qPCR, identified five *TaVPE* genes (*TaVPE6*, *TaVPE15*, *TaVPE18*, *TaVPE25* and *TaVPE26*) that were expressed at significantly higher levels in purple grains than in white grains during the middle and late stages of grain filling. These genes may provide entry points for mechanistic research and for breeding of anthocyanin-rich wheat.

## 1. Introduction

Bread wheat (*Triticum aestivum* L., 2*n* = 6x = 42, AABBDD) is one of the most widely cultivated staple crops worldwide, providing approximately one-fifth of the calories consumed by the global population. The allohexaploid genome of bread wheat originated from two successive rounds of hybridization and polyploidization among three diploid ancestors [1,2], and the release of its fully annotated reference genome, together with recent pan-genome resources, has greatly accelerated gene discovery and functional genomics in this crop [3,4]. Polyploidization and subsequent gene duplication events have profoundly shaped the expansion and functional diversification of gene families in wheat, making it an excellent system for investigating gene family evolution [5].

Coloured-grain wheat, including purple-grained types, accumulates anthocyanins in the grain coat, and these pigments have attracted interest for their antioxidant and health-promoting properties [6,7,8,9], and anthocyanin-biofortified black, blue and purple wheats have demonstrated enhanced nutritional value in derived food products [10,11]. Anthocyanin biosynthesis proceeds through the flavonoid pathway under the transcriptional control of the conserved MBW (MYB–bHLH–WD40) complex [12,13,14,15,16,17]. In purple-pericarp wheat, pigment deposition is governed by *TaPpm1* and *TaPpb1* together with other MYB and bHLH regulators [18,19,20,21,22,23,24,25,26]. The downstream steps that package anthocyanins into vacuoles and coordinate them with grain development, however, remain poorly characterized. Because VPEs are vacuolar proteases central to protein maturation and programmed cell death, they are plausible participants in this process, yet their involvement has not been examined.

Vacuolar processing enzymes (VPEs) are legumain-type (family C13) cysteine proteases that are synthesized as inactive zymogens and are self-activated through sequential autocatalytic processing under acidic conditions [27]. VPEs exhibit caspase-like (YVADase) activity and act as central executors of vacuole-mediated programmed cell death (PCD) in plants [28,29,30]. Functionally, VPEs process seed storage protein precursors in *Arabidopsis* [31] and are required for efficient glutelin processing and proper protein body formation in rice seeds [32,33]; they also participate in seed coat formation during the early stages of seed development [34]. PCD is an integral component of cereal grain development, particularly in the endosperm and aleurone [35,36], and VPEs have been demonstrated to mediate virus-induced hypersensitive cell death [37], mycotoxin-induced cell death [38] and defense responses against pathogens [39,40,41,42]. In addition, VPE expression is up-regulated in lytic vacuoles during senescence and under various stress conditions [43,44,45], and VPE-associated proteolytic networks are closely coupled with ABA-regulated maturation programmes in developing cereal seeds [46]. Collectively, these findings indicate that VPEs are multifunctional proteases linking protein processing, vacuolar dynamics and PCD during seed development.

Gene duplication is a major driving force underlying the expansion and functional innovation of plant gene families [47,48], and the ratio of non-synonymous to synonymous substitution rates (Ka/Ks) is widely used to diagnose the selective pressure acting on duplicated genes [49]. In wheat, genome-wide identification combined with expression profiling has proven to be a powerful strategy for resolving large gene families involved in development and stress adaptation [50]. Although the *VPE* family has been well characterized in *Arabidopsis* and rice, and a genome-wide analysis of the family has recently been reported in soybean [51], a systematic genome-wide analysis of the VPE family in hexaploid wheat is still lacking. Moreover, because anthocyanin accumulation in purple wheat grains proceeds concomitantly with intense vacuolar activity and PCD in the pericarp during grain filling, VPEs represent plausible but completely unexplored players in grain colour formation. Clarifying the composition, evolution and expression of the wheat *VPE* family is therefore essential for understanding grain development and pigmentation and for exploiting coloured-wheat germplasm in breeding.

In this study, we performed a genome-wide identification and comprehensive characterization of the *TaVPE* gene family in hexaploid wheat, including analyses of physicochemical properties, phylogenetic relationships, conserved motifs, protein domains, gene structures, chromosomal distribution, gene duplication and synteny, and promoter *cis*-acting elements. By integrating publicly available expression resources with transcriptome sequencing of grains of the purple-grained winter wheat cultivar Donghei-1 (D1) and the white-grained cultivar Jinmai-66 (J3) at 17, 31, 36 and 41 days after flowering (DAF), followed by RT-qPCR validation, we systematically characterized the expression dynamics of *TaVPE* genes during grain development. This work provides the first comprehensive characterization of the wheat *VPE* family and identifies candidate *TaVPE* genes associated with purple grain colour, laying a foundation for dissecting the molecular mechanisms of grain pigmentation and for the molecular breeding of anthocyanin-rich wheat.

## 2. Materials and Methods

### 2.1. Plant Materials and Sample Collection

The purple-grained winter wheat (*Triticum aestivum* L.) cultivar Donghei-1 (D1) and the white-grained cultivar Jinmai-66 (J3) were grown under identical field conditions during the normal growing season. Individual florets were tagged at anthesis, and developing grains were collected at 17, 31, 36 and 41 days after flowering (DAF), covering the major stages of grain filling and anthocyanin accumulation in the purple cultivar. For each genotype and stage, grains from at least five independent ears were pooled to form one biological replicate, and three independent biological replicates were prepared per genotype per stage, giving 24 libraries in total. All samples were immediately frozen in liquid nitrogen and stored at −80 °C until RNA extraction.

### 2.2. Genome-Wide Identification of TaVPE Genes

Protein sequences of bread wheat were retrieved from the IWGSC RefSeq v1.1 annotation [3]. VPE members were identified by integrating two complementary searches. First, the hidden Markov model (HMM) profile of the Peptidase_C13 domain (PF01650) was retrieved from the Pfam database and used as a query to search the wheat proteome with HMMER v3.3.2 [52] (E-value < 1 × 10^−5^). Second, the four *Arabidopsis* VPE proteins (AtVPEα–δ) and the five rice (*Oryza sativa*) VPE proteins were used as queries in BLASTP 2.12.0+ searches against the wheat proteome [53] (E-value < 1 × 10^−5^). After merging the two sets of hits and removing redundant sequences, all candidate proteins were verified for the presence of the hallmark Peptidase_C13 and legumain_C domains using the NCBI Conserved Domain Database (CDD) [54] and Pfam [55].

### 2.3. Physicochemical Properties and Subcellular Localization Prediction

The physicochemical properties of the TaVPE proteins, including amino acid length, molecular weight (MW), theoretical isoelectric point (pI), instability index, aliphatic index and grand average of hydropathicity (GRAVY), were calculated with the ExPASy ProtParam tool [56] based on the Kyte–Doolittle scale [57]. Subcellular localization was predicted with WoLF PSORT [58].

### 2.4. Phylogenetic Analysis

The full-length sequences of the 27 TaVPE, four AtVPE and five OsVPE proteins were aligned using ClustalW. An unrooted neighbour-joining (NJ) phylogenetic tree was constructed in MEGA 11 [59] with the p-distance substitution model, pairwise deletion of gaps and 1000 bootstrap replicates. The tree was visualized and annotated with the Interactive Tree of Life (iTOL) v6 [60].

### 2.5. Conserved Motifs, Protein Domains and Gene Structure Analysis

Conserved motifs were detected in the TaVPE proteins using the MEME suite [61], with the maximum number of motifs set to 10 and default parameters for motif width. Domain architecture was annotated using the NCBI CDD [54]. Exon–intron structures were displayed by comparing the coding sequences with the corresponding genomic DNA sequences using the Gene Structure Display Server (GSDS) 2.0 [62].

### 2.6. Synteny and Ka/Ks Analysis

The physical positions of *TaVPE* genes on wheat chromosomes were extracted from the IWGSC RefSeq v1.1 annotation file and visualized with TBtools-II [63]. For intraspecific synteny analysis, an all-against-all BLASTP 2.12.0+ search of the wheat proteome [53] was performed, and collinear gene pairs were detected with MCScanX [64]. The non-synonymous (Ka) and synonymous (Ks) substitution rates of each duplicated pair were calculated with KaKs_Calculator [65], and the duplication events were displayed as a Circos plot in TBtools-II [63]. For interspecific synteny analysis, genome sequences and annotation files of *Arabidopsis* thaliana, rice (*Oryza sativa*), maize (*Zea mays*) and foxtail millet (*Setaria italica*) were downloaded from public genome databases, and orthologous VPE gene pairs were identified and visualized using the same MCScanX–TBtools-II pipeline [63,64].

### 2.7. Analysis of Cis-Acting Elements in Promoters

The 2000 bp genomic sequences upstream of the translation start codon of each *TaVPE* gene were extracted from the wheat reference genome with TBtools-II [63] and submitted to the PlantCARE database [66] for *cis*-acting regulatory element prediction. Elements related to hormone response, stress response, light response and transcription factor binding (MYB/bHLH) were counted, and their distribution was visualized with TBtools-II [63].

### 2.8. Gene Expression Profiles Based on WheatOmics

Transcript abundances of *TaVPE* genes across multiple wheat tissues, developmental stages and hormone-treatment samples were obtained from the WheatOmics database v1.0 [67] in the form of transcripts per million (TPM) values. The dataset covered 12 developmental stages (seedling, three-leaf, fifth-leaf, tillering, flag-leaf, full boot, ear emergence, 30% spike, anthesis, milk grain, dough, ripening) and four hormone treatments (DMSO control, 6-BA, ABA, SA). After row Z-score normalization of expression values, a heatmap was constructed using TBtools-II [63].

### 2.9. RNA Sequencing and Transcriptome Analysis

Total RNA was extracted from D1 and J3 grains collected at 17, 31, 36 and 41 DAF (three biological replicates per genotype per stage; 24 libraries in total), and strand-specific libraries were sequenced on an Illumina platform in paired-end 150 bp mode. Raw reads were quality-filtered and trimmed with Trimmomatic v0.39 [68]. Clean reads were aligned to the IWGSC RefSeq v2.1 reference genome [3] using STAR [69]; gene identifiers from the v1.1 annotation were mapped to their v2.1 counterparts using the IWGSC correspondence table, so that the gene identifiers quoted hereafter follow the v2.1 nomenclature. The aligned reads were sorted and indexed with SAMtools [70], and gene-level read counts were quantified with RSEM [71]. Transcript abundance is reported as fragments per kilobase of transcript per million mapped reads (FPKM), which is appropriate for comparing expression between genes within this single tissue type. Differentially expressed genes (DEGs) between D1 and J3 at each stage were identified with DESeq2 v1.36 [72] using the thresholds |log2(fold change)| ≥ 1 and adjusted *p*-value < 0.05. Principal component analysis (PCA) and Venn diagram construction were performed in R.

### 2.10. RT-qPCR Validation

Total RNA was extracted from the same grain samples used for RNA-seq with the UNIQ-10 Column Trizol Total RNA Isolation Kit (B51131, BBI, Shanghai, China), and first-strand cDNA was synthesized with the MightyScript Plus First Strand cDNA Synthesis Master Mix (gDNA digester) (B639252, BBI, Shanghai, China). Gene-specific primers were designed from the coding sequences; specific amplification was obtained for 25 of the 27 *TaVPE* genes, whereas reliable primers could not be obtained for *TaVPE12* and *TaVPE23*, most likely because of the high sequence similarity among their homoeologues and the presence of repetitive regions. qPCR was performed with the 2X HyperMB Universal SYBR Green qPCR Master Mix (B690016) on a QuantStudio 6 Flex Real-Time PCR System (Thermo Fisher Scientific, Waltham, MA, USA), with primers synthesized by Shanghai Sangon Biotech (Shanghai, China). *TaActin* was used as the internal reference gene, and relative expression levels were calculated by the 2^−ΔΔCT^ method [73].

Statistical analyses were performed in GraphPad Prism v10. For the RT-qPCR data, differences between the two cultivars at each developmental stage were assessed by Student’s *t*-test. All *p*-values reported for multiple comparisons are adjusted for multiplicity. Significance markers in figures denote adjusted *p*-values as follows: ns, not significant (*p* ≥ 0.05); * *p* < 0.05; ** *p* < 0.01; *** *p* < 0.001. Data are presented as mean ± SD from at least three independent biological replicates per group.

## 3. Results

### 3.1. Identification and Physicochemical Properties of the TaVPE Family Members

Through the integrated analysis of BLASTP 2.12.0+, HMMER, and NCBI-CDD validation, a total of 27 TaVPE members were identified. The basic physicochemical properties of the 27 identified TaVPE proteins were predicted and analyzed (Table 1 and Table S1). The amino acid sequence lengths of TaVPE members ranged from 361 aa (TaVPE5) to 515 aa (TaVPE1), with corresponding molecular weights varying from 40.43 kDa (TaVPE5) to 56.96 kDa (TaVPE1), indicating that most TaVPE proteins are medium-sized proteins of approximately 40–57 kDa. The theoretical isoelectric points (pI) ranged from 5.14 (TaVPE27) to 7.96 (TaVPE9); except for TaVPE9, which was weakly alkaline (pI > 7), all other members had pI values below 7, suggesting that TaVPE proteins are predominantly acidic. The instability index varied between 37.16 (TaVPE11) and 45.75 (TaVPE21). According to the criterion that a protein with an instability index greater than 40 is regarded as unstable, 20 TaVPE members were predicted to be unstable proteins, whereas only seven members (TaVPE2, TaVPE3, TaVPE9, TaVPE10, TaVPE11, TaVPE17 and TaVPE20) were predicted to be stable. The aliphatic index ranged from 66.63 (TaVPE6) to 84.52 (TaVPE5), reflecting relatively high thermal stability of the TaVPE proteins. All members exhibited negative GRAVY values (−0.544 to −0.237), indicating that the TaVPE family proteins are hydrophilic. Subcellular localization prediction with WoLF PSORT showed that the majority of TaVPE proteins (21 of the 27 members) were assigned to the extracellular/secretory category. Because WoLF PSORT infers localization from N-terminal targeting signals, this assignment most likely reflects signal-peptide-dependent entry into the ER-Golgi secretory route rather than the final destination of the mature protein; it is therefore consistent with the routing of VPE zymogens towards the vacuole, in which the mature enzyme is activated. Six members (TaVPE5, TaVPE6, TaVPE7, TaVPE10, TaVPE11 and TaVPE27) were predicted to localize in the cytosol, and TaVPE5 was predicted in both the mitochondria and the cytosol, as listed in Table 1. Taken together, the *TaVPE* family members share similar physicochemical characteristics but differ in their predicted subcellular distribution, implying potential functional diversification among family members.

### 3.2. Phylogenetic Analysis of VPE Proteins in Wheat, Arabidopsis and Rice

**Figure 1:**
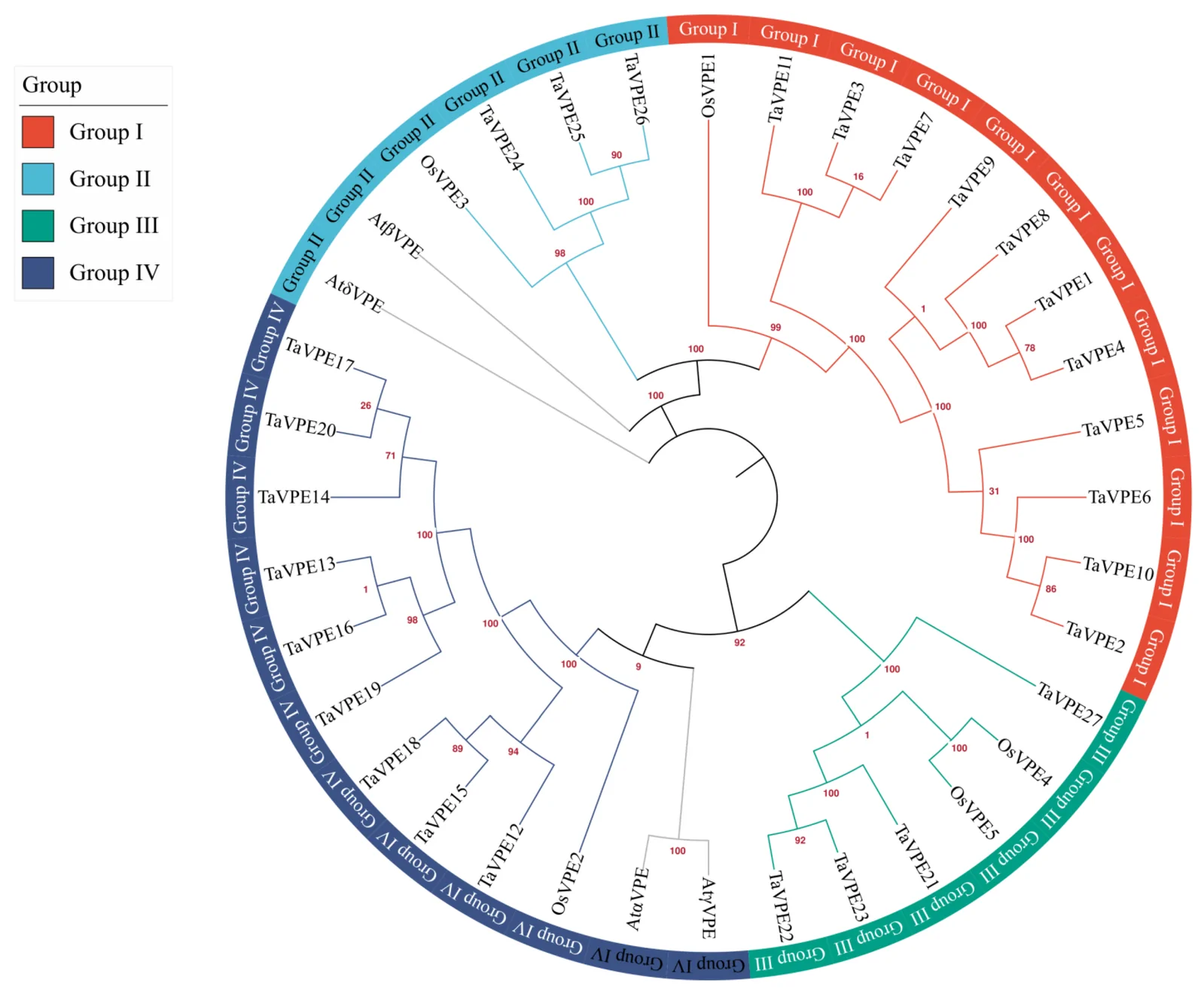
Phylogenetic tree of VPE proteins from wheat, *Arabidopsis* and rice.

To investigate the evolutionary relationships among VPE family members, a phylogenetic tree was constructed using the full-length amino acid sequences of 27 TaVPE proteins from wheat (*Triticum aestivum*), 4 AtVPE proteins from *Arabidopsis thaliana* and 5 OsVPE proteins from rice (Oryza sativa) (Figure 1, Table S2). Based on the phylogenetic topology, the 36 VPE proteins were classified into four distinct groups (Group I–IV). Group I was the largest clade, containing 12 members, including 11 TaVPE proteins (TaVPE1–TaVPE11) and OsVPE1, but no *Arabidopsis* VPE. Group II consisted of 6 members, comprising three TaVPEs (TaVPE24, TaVPE25 and TaVPE26), OsVPE3, and two *Arabidopsis* VPEs (AtβVPE and AtδVPE). Group III contained 6 members, including four TaVPEs (TaVPE21, TaVPE22, TaVPE23 and TaVPE27) and two rice VPEs (OsVPE4 and OsVPE5), with no *Arabidopsis* member. Group IV harbored 12 members, including nine TaVPEs (TaVPE12–TaVPE20), OsVPE2, and two *Arabidopsis* VPEs (AtαVPE and AtγVPE). Notably, TaVPE proteins were present in all four groups but were unevenly distributed, with Groups I and IV being the predominant clades. The VPE proteins from the three species were intermixed within the same groups, and most TaVPEs clustered more closely with rice OsVPEs than with *Arabidopsis* AtVPEs, which is consistent with the closer phylogenetic relationship between wheat and rice as monocots. These results suggest that the VPE gene family is evolutionarily conserved across monocots and dicots, and that the expansion of the *TaVPE* family in wheat mainly occurred in Groups I and IV, probably as a consequence of polyploidization events during wheat evolution.

### 3.3. Phylogenetic Relationship, Conserved Motif, Domain Architecture and Gene Structure Analyses of the TaVPE Family

**Figure 2:**
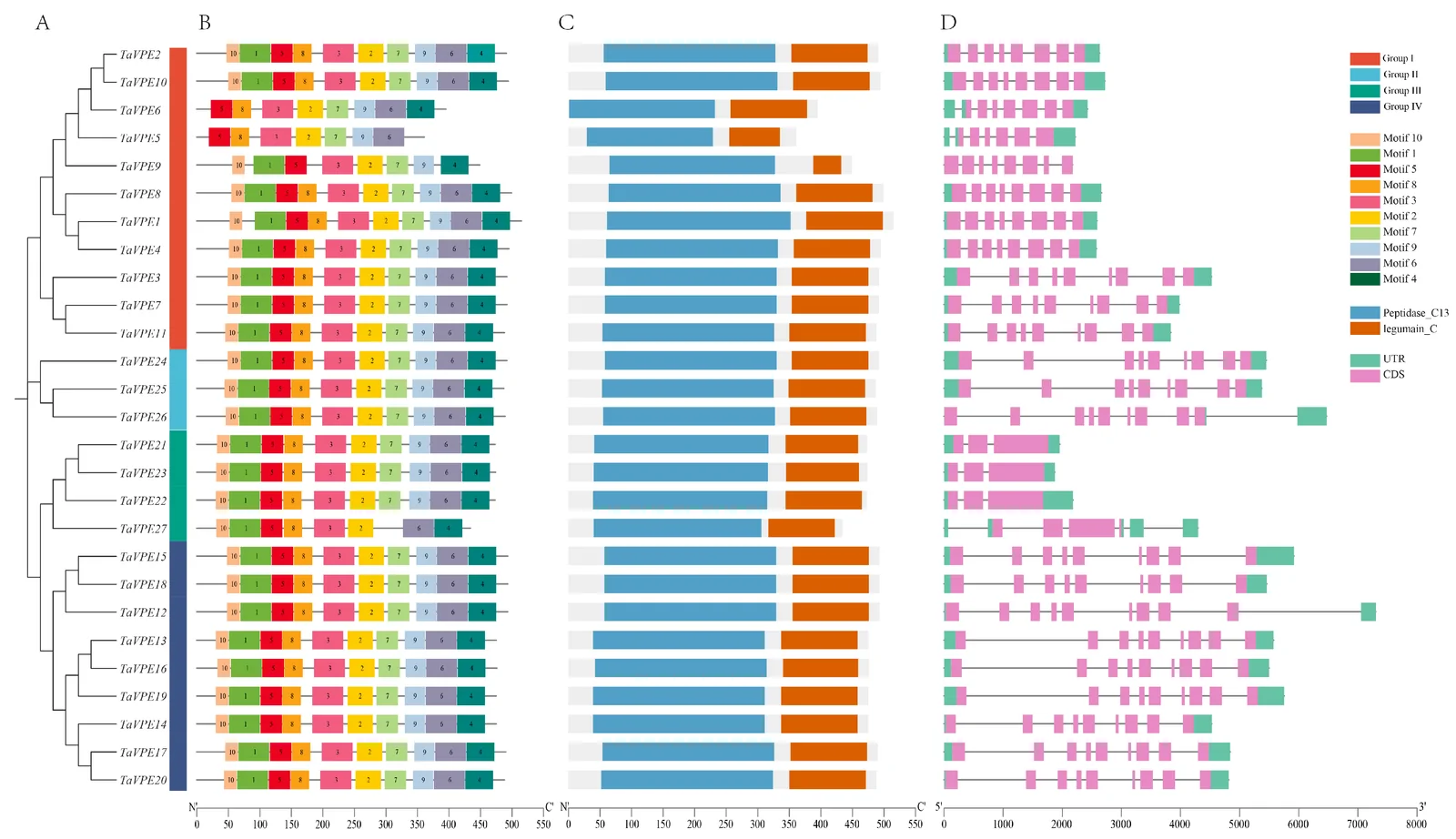
Phylogenetic relationship, conserved motif composition, protein domain architecture and gene structure of the *TaVPE* family in wheat. (**A**) Phylogenetic tree of the 27 TaVPE proteins constructed by the neighbour-joining method with 1000 bootstrap replicates. (**B**) Distribution of ten conserved motifs (motif 1–10) identified by MEME analysis. (**C**) Conserved domain architecture of TaVPE proteins. (**D**) Exon–intron organization of the *TaVPE* genes.

To further characterize the *TaVPE* gene family, an intraspecific phylogenetic tree of the 27 TaVPE proteins was constructed, and their conserved motifs, protein domains and gene structures were analyzed in an integrated manner (Figure 2). Consistent with the phylogenetic analysis of VPEs from wheat, *Arabidopsis* and rice, the 27 TaVPE proteins were clustered into four groups, with 11, 3, 4 and 9 members in Groups I, II, III and IV, respectively (Figure 2A).

A total of ten conserved motifs (motif 1–10) were identified in the TaVPE proteins by MEME analysis (Figure 2B). The vast majority of TaVPE members contained all ten motifs, and the type, order and number of motifs were highly similar among members within the same group, which strongly supported the reliability of the phylogenetic grouping. Only a few members showed motif loss: TaVPE6 lacked motif 1 and motif 10 at the N-termini, and TaVPE5 retained only seven motifs (lacking motifs 1, 4 and 10), suggesting possible functional divergence of these members.

Protein domain prediction showed that all 27 TaVPE proteins harbored the two hallmark domains of the VPE/legumain family, namely the N-terminal Peptidase_C13 catalytic domain and the C-terminal legumain_C domain (Figure 2C). The position and length of the two domains were highly conserved across the family, although several members (e.g., TaVPE5 and TaVPE6) possessed relatively short N-terminal regions, which was consistent with the loss of N-terminal motifs observed in the motif analysis.

Gene structure analysis revealed considerable divergence in exon–intron organization among *TaVPE* genes (Figure 2D). The number of exons ranged from three to nine: the four Group III members (*TaVPE21*, *TaVPE22*, *TaVPE23* and *TaVPE27*) contained only three or four exons and were nearly intronless, whereas Group II members (*TaVPE24*, *TaVPE25* and *TaVPE26*) and Group IV members possessed nine exons. In addition, the Group II and Group IV members spanned longer genomic regions with extended introns. Generally, closely related members within the same phylogenetic group exhibited similar exon–intron patterns, further corroborating the phylogenetic classification. Most *TaVPE* genes contained 5′ or 3′ untranslated regions (UTRs), whereas UTRs were not detected at one or both ends in a few members (e.g., *TaVPE9*, *TaVPE26*). Taken together, the high conservation of motif composition, domain architecture and exon–intron organization within groups, together with the structural divergence between groups, indicates that *TaVPE* members within the same group may perform similar functions, while structural diversification among groups may underlie their functional differentiation.

### 3.4. Chromosomal Distribution of the TaVPE Genes

**Figure 3:**
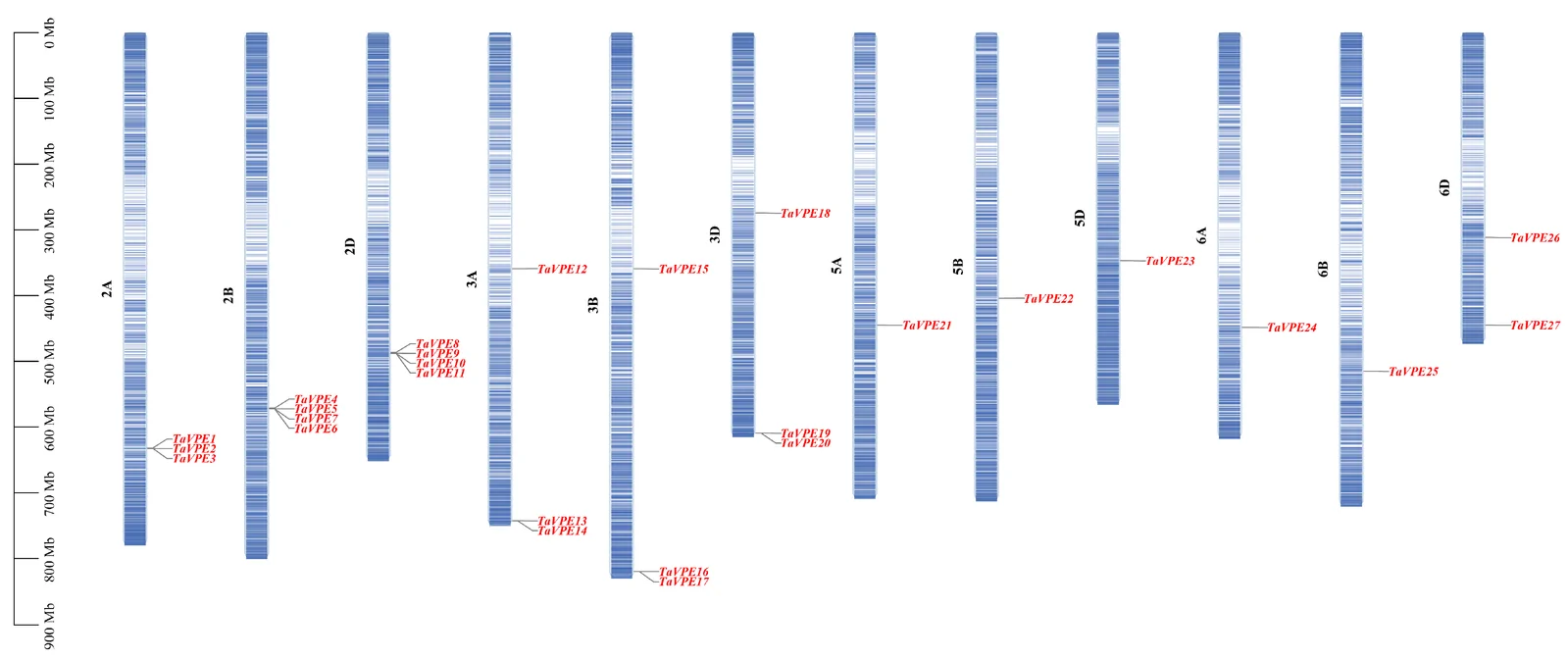
Chromosomal distribution of *TaVPE* genes in wheat. The scale bar represents the physical distance (Mb); gene names are shown in red.

Based on the wheat reference genome annotation, the 27 *TaVPE* genes were mapped onto the chromosomes of wheat (Figure 3). The number of *TaVPE* genes per chromosome ranged from one to four: chromosomes 2B and 2D each harbored four genes (*TaVPE4-TaVPE7* and *TaVPE8-TaVPE11*, respectively); chromosomes 2A, 3A, 3B and 3D each contained three genes (*TaVPE1-TaVPE3*, *TaVPE12-TaVPE14*, *TaVPE15-TaVPE17* and *TaVPE18-TaVPE20*, respectively); chromosome 6D contained two genes (*TaVPE26* and *TaVPE27*); and chromosomes 5A, 5B, 5D, 6A and 6B each carried only one gene (*TaVPE21*, *TaVPE22*, *TaVPE23*, *TaVPE24* and *TaVPE25*, respectively). These per-chromosome counts are identical to those listed in Table 1 and Table S2, and the 27 genes are distributed across 12 chromosomes in total. The *TaVPE* genes were unevenly distributed on these 12 chromosomes, which belong to four homoeologous groups (groups 2, 3, 5 and 6), while no *TaVPE* member was found on the chromosomes of homoeologous groups 1, 4 and 7. Among the three subgenomes, 8, 9 and 10 *TaVPE* genes were distributed in the A, B and D subgenomes, respectively, indicating a relatively balanced distribution among subgenomes. Notably, *TaVPE* genes on the same chromosome tended to form clusters: *TaVPE1-TaVPE3*, *TaVPE4-TaVPE7* and *TaVPE8-TaVPE11* formed three gene clusters on chromosomes 2A, 2B and 2D, respectively, and *TaVPE12-TaVPE14*, *TaVPE15-TaVPE17* and *TaVPE18-TaVPE20* were located at the distal ends of chromosomes 3A, 3B and 3D, respectively. These clustered genes, together with the one-to-one correspondence of *TaVPE* members across the A, B and D homoeologous chromosomes, suggest that the expansion of the *TaVPE* family in wheat was mainly driven by segmental duplication associated with polyploidization, accompanied by tandem duplication events on chromosomes of groups 2 and 3.

### 3.5. Intraspecific Synteny and Ka/Ks Analysis of the TaVPE Genes

**Figure 4:**
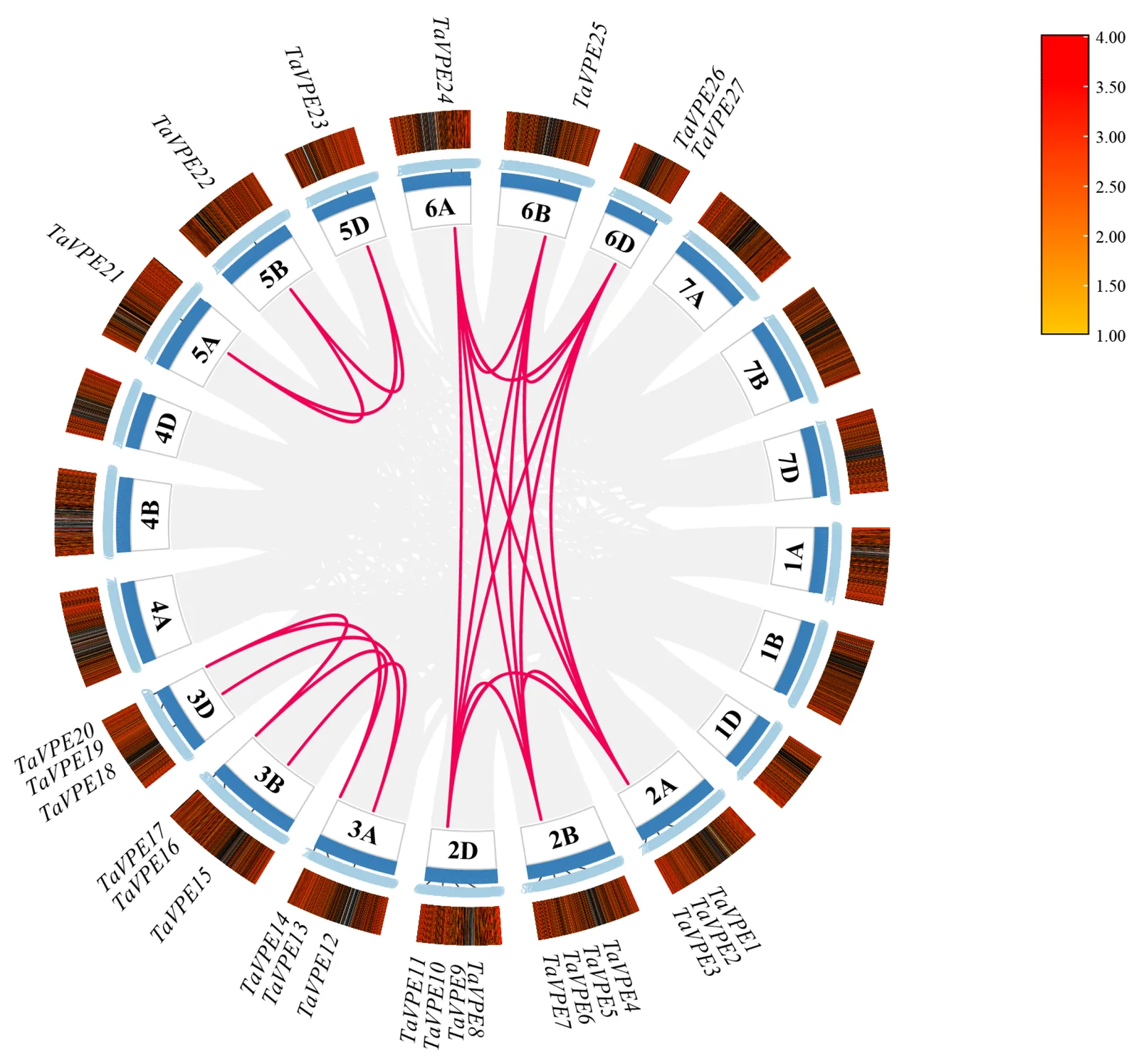
Intraspecific synteny analysis of *TaVPE* genes in wheat. Gray lines indicate syntenic blocks within the wheat genome, and red lines indicate syntenic relationships among *TaVPE* genes.

To elucidate the duplication and expansion mechanism of the *TaVPE* gene family, intraspecific synteny analysis was performed in the wheat genome (Figure 4). Intraspecific synteny analysis identified 26 collinear *TaVPE* gene pairs, here defined as the number of pairwise *TaVPE* synteny blocks detected by MCScanX in the hexaploid wheat genome; these were distributed across chromosomes of homoeologous groups 2, 3, 5 and 6. Most of the collinear pairs were located on homoeologous chromosomes of the A, B and D subgenomes, such as *TaVPE1*/*TaVPE4* (2A–2B), *TaVPE1*/*TaVPE8* (2A–2D), *TaVPE2*/*TaVPE5* (2A–2B), *TaVPE12*/*TaVPE15* (3A–3B), *TaVPE12*/*TaVPE18* (3A–3D), *TaVPE16*/*TaVPE19* (3B–3D), *TaVPE21*/*TaVPE22*/*TaVPE23* (5A–5B–5D) and *TaVPE24*/*TaVPE25*/*TaVPE26* (6A–6B–6D), indicating that segmental duplication arising from wheat polyploidization was the major driving force for the expansion of the *TaVPE* family. In addition, several collinear pairs were detected between non-homoeologous chromosomes, such as the pairs between group 2 chromosomes and chromosome 6 (e.g., *TaVPE3*/*TaVPE24*, *TaVPE4*/*TaVPE25* and *TaVPE5*/*TaVPE26*), suggesting that ancient segmental duplication events also contributed to family expansion.

To assess the selective pressure acting on the duplicated *TaVPE*, the Ka/Ks ratios of all 40 duplicated gene pairs listed in Table S3 were less than 1, ranging from 0.0596 to 0.4657. The *TaVPE13*/*TaVPE16* pair exhibited the lowest ratio (0.0596) and the *TaVPE5*/*TaVPE9* pair the highest (0.4657). Because all ratios were markedly below the neutral expectation of Ka/Ks = 1 (the highest value being less than half of 1), and because the Ks values remained well below the saturation threshold, these results indicate that the *TaVPE* gene family has evolved under purifying selection, which acts to maintain the functional conservation of *TaVPE* genes after duplication. The ratios were interpreted against the neutral value of Ka/Ks = 1; formal *p*-values against a neutral null model were not computed. The breadth of the range (approximately 7.8-fold) suggests that the strength of constraint is not uniform across paralogues: *TaVPE5/TaVPE9 (0.4657)* has experienced the weakest constraint and may have accumulated more amino-acid substitutions, whereas *TaVPE13*/*TaVPE16* (0.0596) is the most strongly constrained pair.

### 3.6. Interspecific Synteny Analysis Between Wheat and Other Species

**Figure 5:**
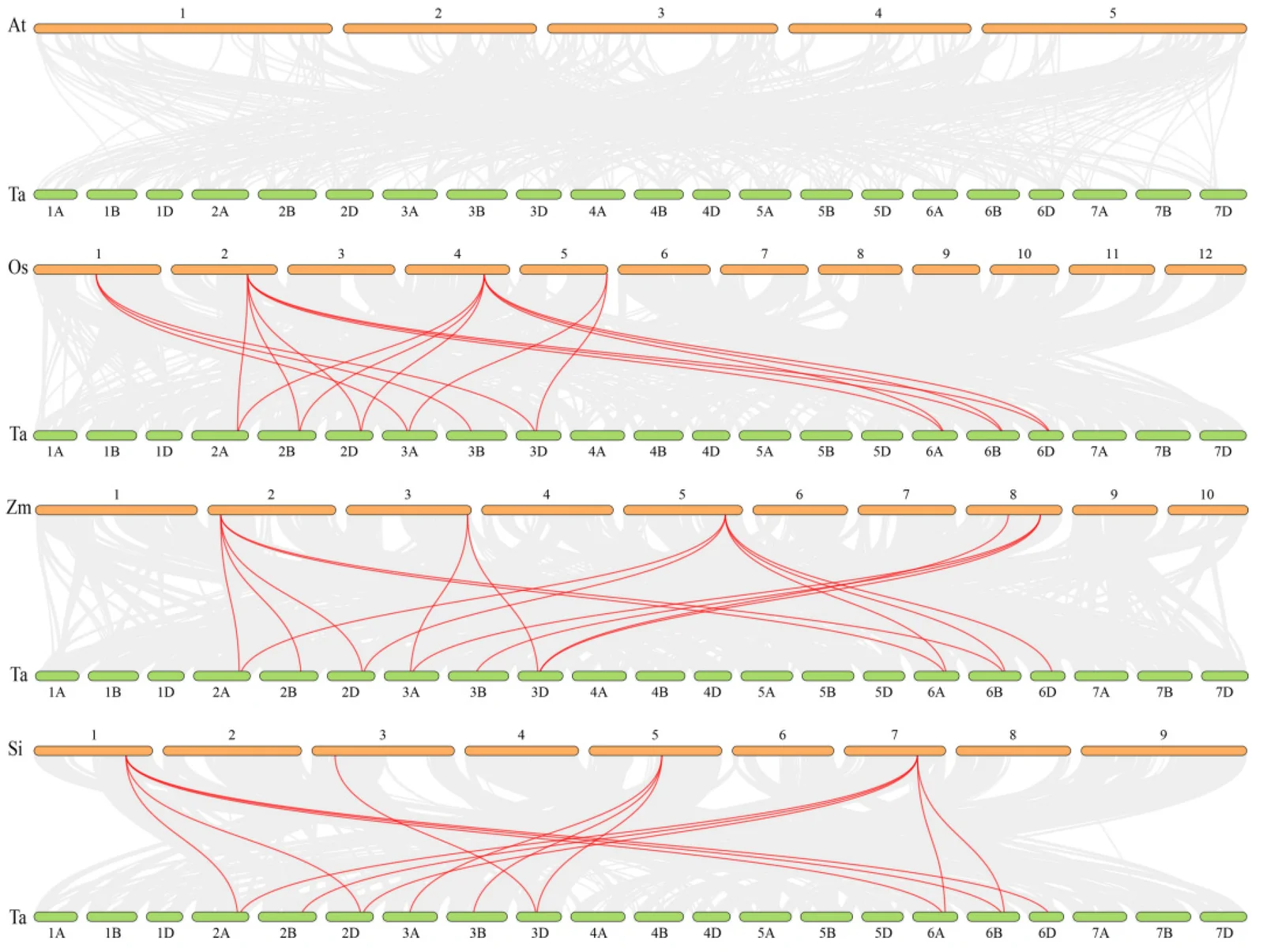
Synteny between wheat (Ta) and *Arabidopsis thaliana* (At), *Oryza sativa* (Os), *Zea mays* (Zm) and *Setaria italica* (Si). Gray lines indicate interspecific syntenic blocks, and red lines indicate syntenic *VPE* gene pairs.

To further investigate the evolutionary origin and conservation of the *TaVPE* gene family, interspecific synteny analysis was performed between wheat and four representative species, including the dicot *Arabidopsis thaliana* and the monocots *Oryza sativa*, *Zea mays* and *Setaria italica* (Figure 5; Table S4). A total of 47 orthologous *VPE* gene pairs were identified, comprising 17 pairs between wheat and rice, 16 pairs between wheat and maize, and 14 pairs between wheat and foxtail millet, whereas no collinear *VPE* gene pair was detected between wheat and *Arabidopsis*. This distribution pattern is consistent with the closer phylogenetic relationship among monocot species.

The collinear *TaVPE* genes were all located on wheat chromosomes of homoeologous groups 2, 3 and 6, and nine *TaVPE* genes participated in the interspecific syntenic relationships. Notably, each *TaVPE* gene was collinear with two *VPE* loci in each of the three monocot species. The one-to-two correspondence between *TaVPE* genes and their monocot orthologs suggests that these *VPE* loci originated from ancient genome duplication events that occurred before the divergence of the *Poaceae* species, and that the syntenic *VPE* loci have been highly conserved during monocot evolution. Moreover, the A, B and D homoeologous copies in wheat were all retained in the syntenic blocks, further confirming that polyploidization played a major role in the expansion of the *TaVPE* family.

### 3.7. Cis-Acting Element Analysis in the Promoters of TaVPE Genes

**Figure 6:**
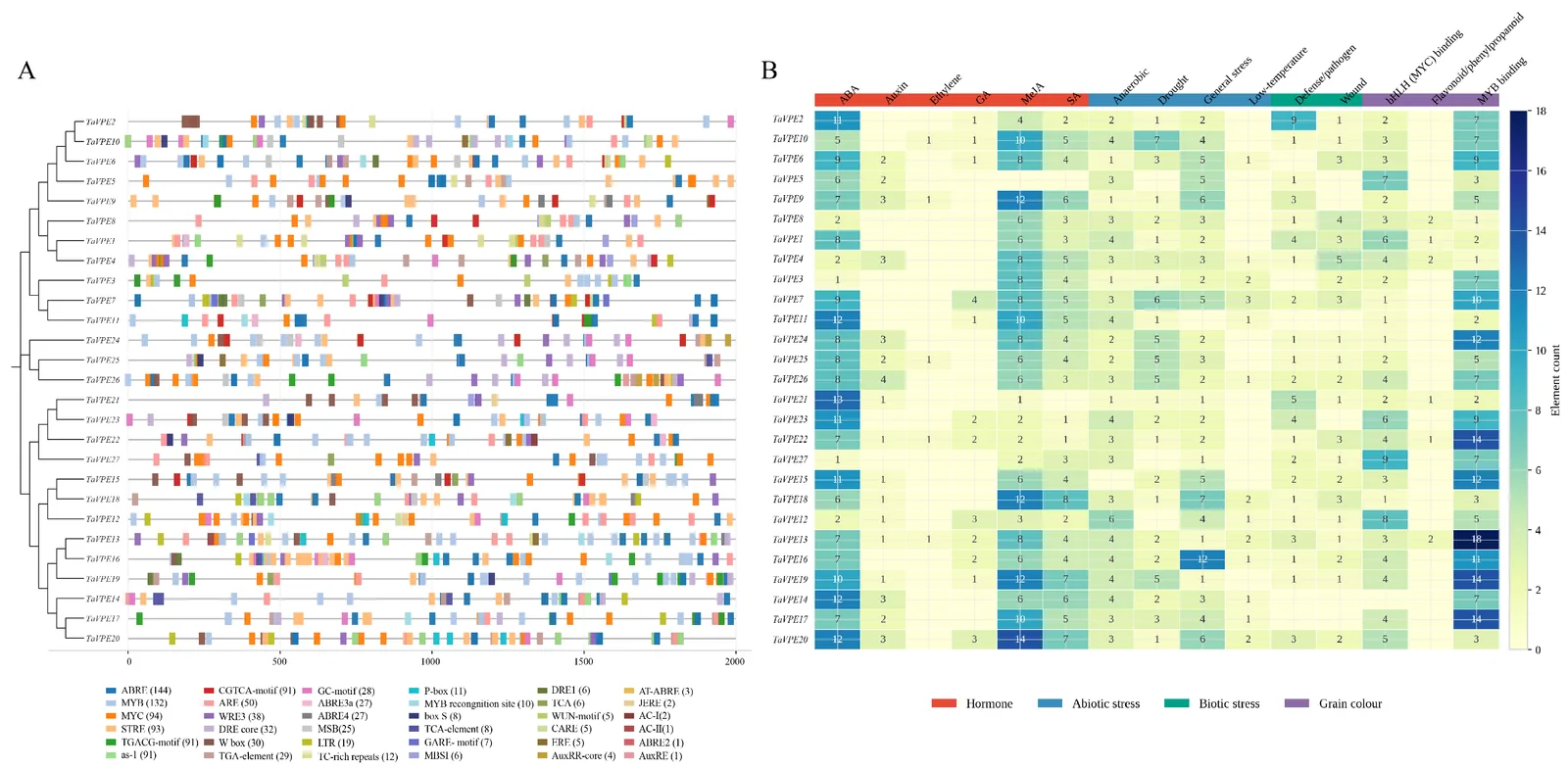
Analysis of *cis*-acting elements in the promoter regions of *TaVPE* genes. (**A**) Distribution of *cis*-acting elements in the 2000 bp upstream promoter regions of the 27 *TaVPE* genes. The phylogenetic tree of the TaVPE proteins is shown on the left, and different colored boxes represent different *cis*-acting elements, with the corresponding element names and numbers listed in the legend below. The scale bar indicates the promoter length (bp). (**B**) Heatmap of the numbers of *cis*-acting elements in each functional category for each *TaVPE* gene. The colored bar at the top indicates the four functional categories: hormone-responsive (red), abiotic stress-responsive (blue), biotic stress-responsive (green) and grain colour-related (purple) elements. Color intensity in the heatmap represents the number of elements.

To explore the potential regulatory mechanisms of *TaVPE* genes, *cis*-acting elements in the 2000 bp upstream promoter regions of the 27 *TaVPE* genes were predicted (Figure 6A). A total of 1144 *cis*-acting elements were identified and classified into four functional categories: hormone-responsive, abiotic stress-responsive, biotic stress-responsive and grain colour-related elements (Figure 6B, Table S5).

Hormone-responsive elements constituted the largest category (553 elements, 48.3%), among which abscisic acid (ABA)-responsive elements (ABREs) were the most abundant (202 elements), followed by methyl jasmonate (MeJA)-responsive elements (CGTCA-motif and TGACG-motif; 184) and salicylic acid (SA)-responsive elements (TCA-element; 105), whereas auxin-(34), gibberellin-(23) and ethylene-responsive (5) elements were relatively scarce. Notably, ABA-responsive elements were present in the promoters of all 27 *TaVPE* genes, suggesting that ABA signaling may play a central role in the regulation of *TaVPE* expression.

Abiotic stress-responsive elements (253 elements) mainly included general stress-responsive elements (STRE; 93), anaerobic-responsive elements (ARE; 78), drought-responsive elements (DRE core/MBS; 63) and low-temperature-responsive elements (LTR; 19). Biotic stress-responsive elements (93) consisted of defense/pathogen-responsive (TC-rich repeats/W-box; 50) and wound-responsive elements (WUN-motif; 43). In addition, a considerable number of elements associated with grain colour formation were detected (245), comprising MYB binding sites (142) involved in flavonoid biosynthesis, bHLH/MYC binding sites (94) and flavonoid- or phenylpropanoid-related elements (9), implying potential roles of *TaVPE* genes in grain pigment accumulation.

The number of cis-elements varied among *TaVPE* members, ranging from 26 (*TaVPE26*) to 57 (*TaVPE19*), and members within the same phylogenetic group generally exhibited similar element compositions. Taken together, these results suggest that *TaVPE* genes are widely involved in hormone signaling, abiotic and biotic stress responses, and grain colour regulation in wheat, with ABA- and MeJA-mediated pathways likely being the predominant regulatory routes.

### 3.8. Expression Profiling of TaVPE Genes in Different Tissues and Under Hormone Treatments

**Figure 7:**
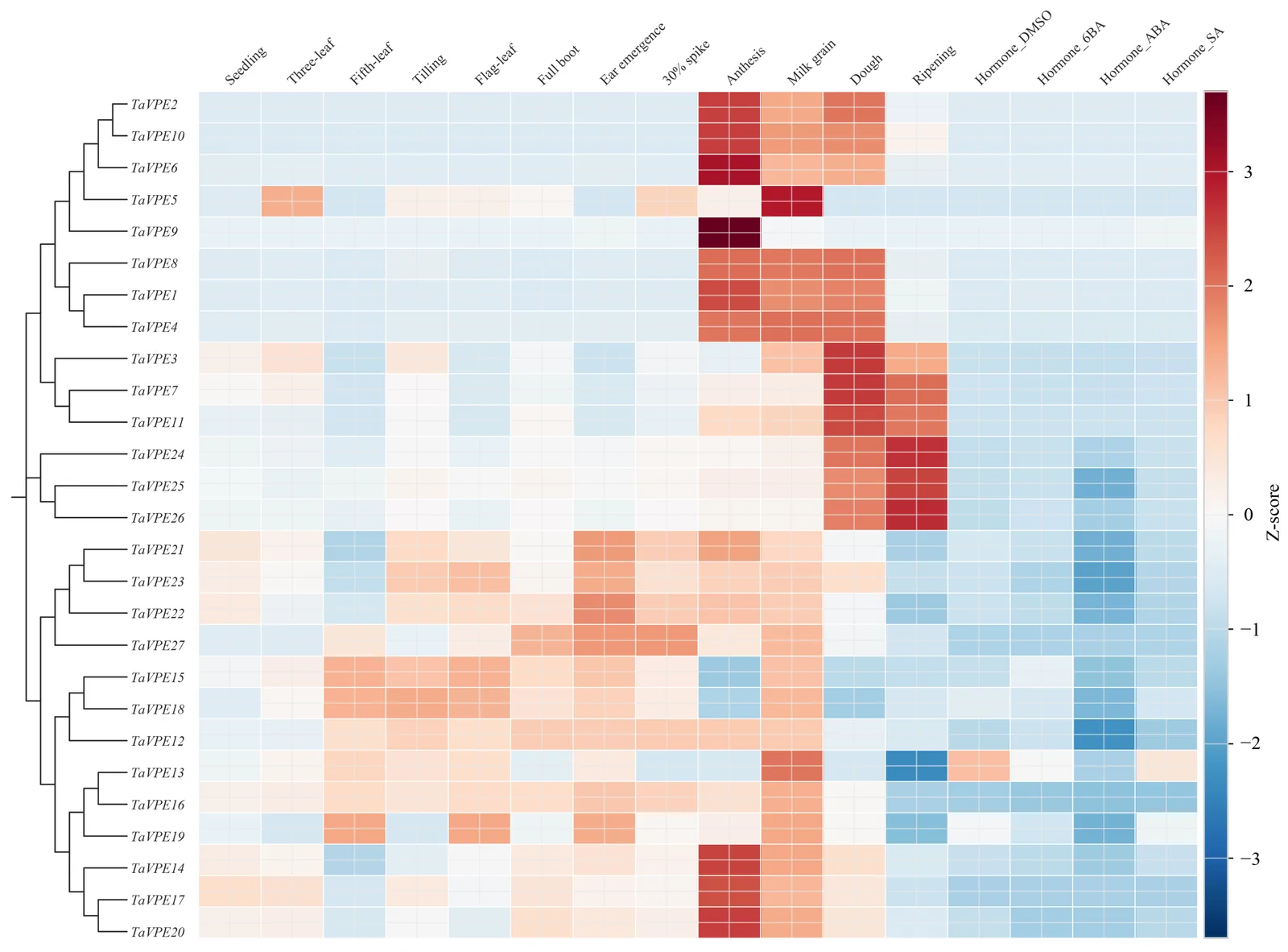
Expression profiles of *TaVPE* genes in different tissues and under hormone treatments. The expression data of 27 *TaVPE* genes in 12 wheat tissues/developmental stages and under hormone treatments were retrieved from the WheatOmics database. Expression values were normalized by Z-score, and the heatmap was generated with hierarchical clustering of the genes. Red and blue indicate relatively high and low expression levels, respectively.

To investigate the expression patterns of *TaVPE* genes, the transcriptomic data of 27 *TaVPE* genes across 12 developmental stages/tissues and under hormone treatments were retrieved from the WheatOmics database (Figure 7, Table S6). The *TaVPE* genes exhibited distinct spatiotemporal expression patterns and were roughly divided into four expression clusters, which largely corresponded to the phylogenetic groups. The eight Group I members (*TaVPE1*, *TaVPE2*, *TaVPE4*, *TaVPE5*, *TaVPE6*, *TaVPE8*, *TaVPE9* and T*aVPE10*) were barely expressed in vegetative tissues but were strongly and specifically induced at anthesis and during grain filling (milk grain and dough stages), with Z-scores ranging from 1.4 to 3.7 at anthesis, suggesting their important roles in reproductive development and grain formation. *TaVPE3*, *TaVPE7* and *TaVPE11* showed a similar but weaker trend, peaking at the dough stage. The Group II members (*TaVPE24*, *TaVPE25* and *TaVPE26*) were preferentially expressed during the middle and late grain-filling stages (milk grain, dough and ripening), with *TaVPE26* reaching its maximum at ripening (Z-score 2.73), indicating potential functions in grain maturation. In contrast, the Group III members (*TaVPE21*, *TaVPE22*, *TaVPE23* and *TaVPE27*) were mainly expressed in vegetative tissues such as tillering, flag-leaf and booting stages, whereas the Group IV members displayed broader expression patterns, being moderately expressed in both vegetative and reproductive tissues, with *TaVPE14*, *TaVPE17* and *TaVPE20* showing relatively high expression at anthesis.

Under hormone treatments, the expression of most *TaVPE* genes was down-regulated to varying degrees compared with the DMSO control, while Group I members were barely responsive. Among the three hormones, ABA treatment caused the most pronounced repression, particularly for *TaVPE12*, *TaVPE13*, *TaVPE23* and *TaVPE25.* The expression of *TaVPE* genes during anthesis and grain development is consistent with the canonical role of VPEs in programmed cell death and seed maturation.

### 3.9. Transcriptome Overview of Developing Purple- and White-Grained Wheat Grains

To provide a whole-transcriptome framework for *TaVPE* expression, we profiled global transcriptional changes between the purple-grained winter wheat cultivar Donghei-1 (D1) and the white-grained cultivar Jinmai-66 (J3) during grain development (17, 31, 36 and 41 days after flowering, DAF; three biological replicates per genotype per stage; 24 libraries in total).

**Figure 8:**
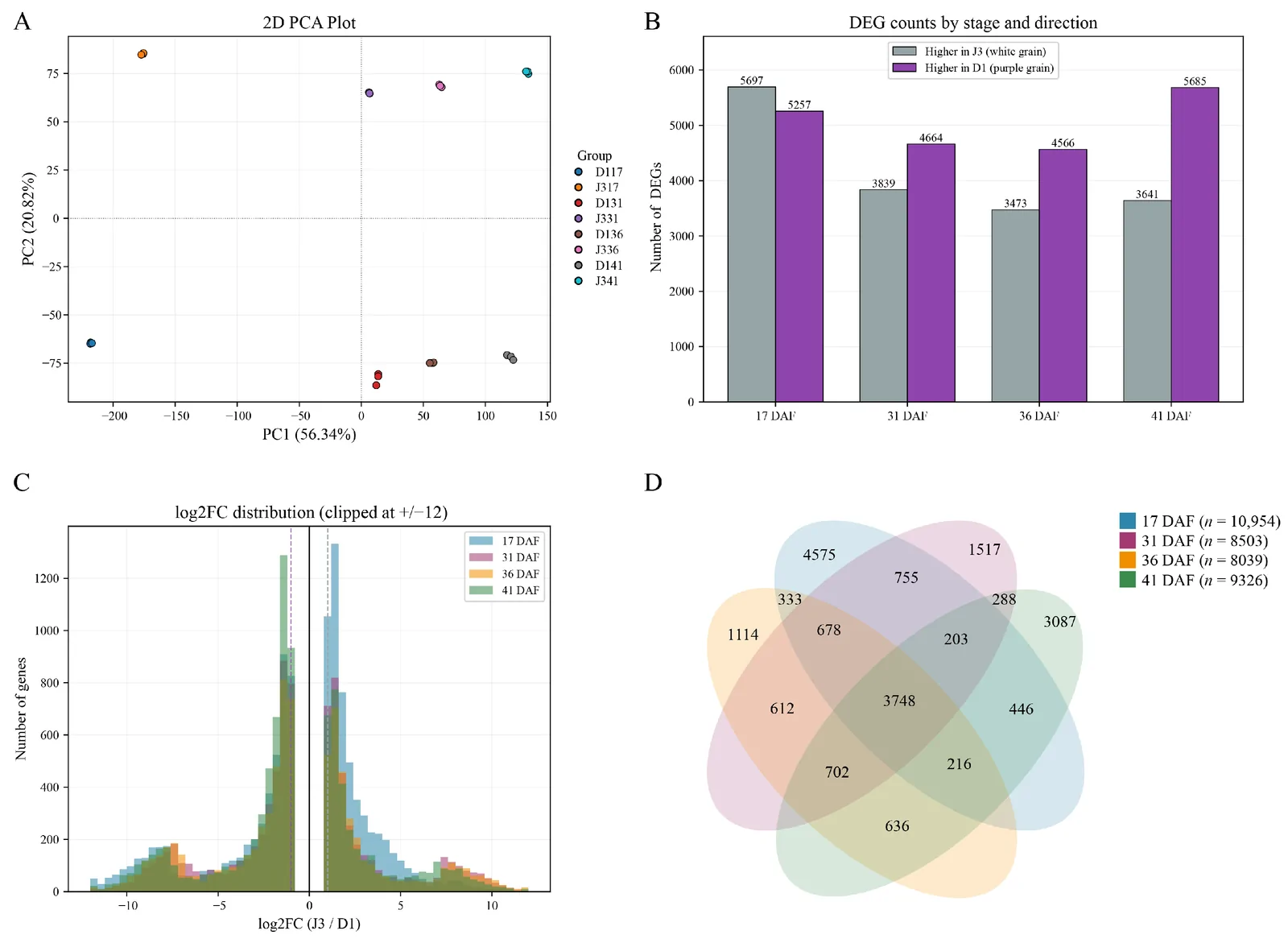
Overview of the grain transcriptomes of the purple-grained wheat cultivar Donghei-1 (D1) and the white-grained cultivar Jinmai-66 (J3) at four developmental stages. (**A**) Principal component analysis (PCA) of the 24 RNA-seq libraries. The percentages of variance explained by PC1 and PC2 are shown on the axes. D117–D141 and J317–J341 represent D1 and J3 samples at 17, 31, 36 and 41 days after flowering (DAF), respectively. (**B**) Numbers of differentially expressed genes (DEGs) between D1 and J3 at each developmental stage. Grey and purple bars indicate DEGs with higher expression in J3 (white grain) and D1 (purple grain), respectively. (**C**) Distribution of log2 fold-change (log2FC, J3/D1) values of DEGs at the four stages (values clipped at ±12); dashed lines indicate the threshold of |log2FC| = 1. (**D**) Venn diagram showing the overlap of DEGs among the four developmental stages; the total number of DEGs at each stage is given in parentheses.

Principal component analysis (PCA) showed that the first two principal components explained 56.34% (PC1) and 20.82% (PC2) of the total variance, accounting for 77.16% cumulatively (Figure 8A). The three biological replicates of each sample clustered tightly together, indicating high reproducibility and reliability of the sequencing data. Samples were separated mainly along PC1 according to the developmental stage, with the 17 DAF samples positioned farthest from the later stages, suggesting that grain developmental stage was the predominant source of transcriptomic variation; meanwhile, the two genotypes were clearly distinguished at each stage.

Differentially expressed genes (DEGs) between D1 and J3 were identified at each stage (Figure 8B). A total of 10,954, 8503, 8039 and 9326 DEGs were detected at 17, 31, 36 and 41 DAF, respectively. At 17 DAF, more DEGs showed higher expression in J3 than in D1 (5697 vs. 5257), whereas at the three later stages the number of DEGs with higher expression in the purple-grained D1 exceeded that in J3 (4664 vs. 3839 at 31 DAF; 4566 vs. 3473 at 36 DAF; 5685 vs. 3641 at 41 DAF), with the largest difference at 41 DAF. The log2 fold-change (log2FC) values of the DEGs displayed an approximately symmetric distribution at all four stages, and most DEGs showed moderate fold changes (Figure 8C). Venn analysis identified 3748 DEGs shared by all four stages, representing a core set of genes that were continuously differentially expressed between the purple- and white-grained cultivars during grain development, whereas 4575, 1517, 1114 and 3087 DEGs were unique to 17, 31, 36 and 41 DAF, respectively (Figure 8D). Because the remaining genes were shared by two or three stages only, the sum of these category sizes is smaller than the total; after de-duplication, 18,910 non-redundant DEGs were identified across the four stages. These results indicate that the transcriptional divergence between D1 and J3 was stage-dependent and became increasingly biased toward genes with higher expression in the purple-grained cultivar as grain development progressed, providing a global transcriptomic basis for subsequent analysis of *TaVPE* expression during grain colour formation.

### 3.10. Expression Analysis of TaVPE Genes During Grain Development

**Figure 9:**
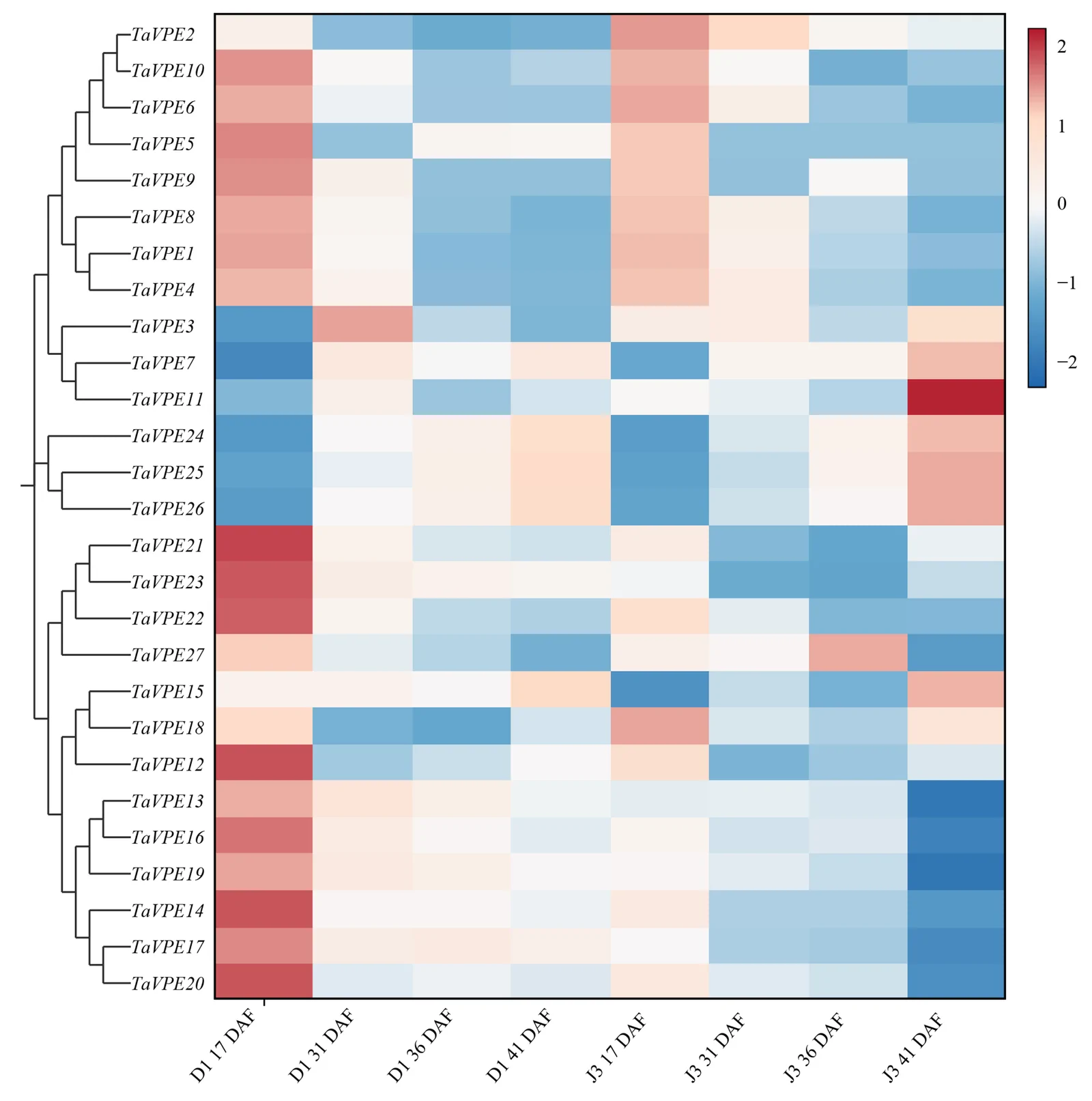
Expression heatmap of the 27 *TaVPE* genes in developing grains of D1 and J3. Expression levels (FPKM) of *TaVPE* genes were extracted from the transcriptome data of grains of the D1 (purple-grained Donghei-1) and J3 (white-grained Jinmai-66) at 17, 31, 36 and 41 days after flowering (DAF), with three biological replicates per genotype per stage. Expression values were normalized by Z-score across samples, and genes were clustered hierarchically. Red and blue indicate relatively high and low expression levels, respectively.

To identify *TaVPE* members potentially associated with grain colour formation, the expression levels (FPKM) of the 27 *TaVPE* genes in the developing grains of D1 (purple grain) and J3 (white grain) at 17, 31, 36 and 41 DAF were extracted from the transcriptome data, and a heatmap was generated using Z-score-normalized expression values (Figure 9; Table S7).

The *TaVPE* genes showed distinct expression patterns during grain development and could be divided into three major expression clusters. The first cluster (including *TaVPE1*, *TaVPE2*, *TaVPE4*, *TaVPE5*, *TaVPE6*, *TaVPE8*, *TaVPE9*, *TaVPE10* and most Group IV members) exhibited the highest expression at 17 DAF, with expression declining progressively as grain development proceeded. The second cluster (*TaVPE24*, *TaVPE25* and *TaVPE26*) showed low expression at the early stage but was strongly induced during the middle and late grain-filling stages, reaching the maximum at 41 DAF. The third cluster (*TaVPE3*, *TaVPE7* and *TaVPE11*) displayed increasing expression toward the late developmental stages. Notably, *TaVPE5*, *TaVPE9* and *TaVPE27* were barely expressed in grains at any stage (FPKM ~0), suggesting that they may not be active during grain development.

Comparison between the two cultivars revealed marked genotype-dependent differences. *TaVPE24* and *TaVPE25* were expressed at substantially higher levels in the purple-grained D1 than in J3 during the middle and late stages: *TaVPE24* showed FPKM values of 60.43–72.43 in D1 versus only 4.44–4.47 in J3 at 36 DAF, and 147.35–154.19 versus 6.16–7.24 at 41 DAF (more than 15- and 20-fold higher in D1, respectively); *TaVPE*25 was approximately 3-fold higher in D1 than in J3 at 36 and 41 DAF (82.89–92.77 vs. 32.82–38.74 and 183.39–194.84 vs. 61.40–63.66). Similarly, *TaVPE4* was expressed at markedly higher levels in D1 than in J3 at 36 DAF (30.89–37.01 vs. 1.27–2.34). In contrast, *TaVPE7* and *TaVPE11* were more highly expressed in the white-grained J3 at 41 DAF (approximately 2- and 3-fold, respectively), while most Group IV members were slightly higher in D1 at 17 DAF. *TaVPE25* qualified as a candidate gene on the strength of both its RNA-seq and RT-qPCR data. *TaVPE24* and *TaVPE4*, although strongly D1-biased in the RNA-seq comparison, were not retained as candidates because their RT-qPCR differences did not reach statistical significance. The five genes retained as candidates on the basis of significant RT-qPCR evidence are *TaVPE6*, *TaVPE15*, *TaVPE18*, TaV*P*E25 and *TaVPE26*.

### 3.11. RT-qPCR Validation of TaVPE Gene Expression in D1 and J3 Grains

**Figure 10:**
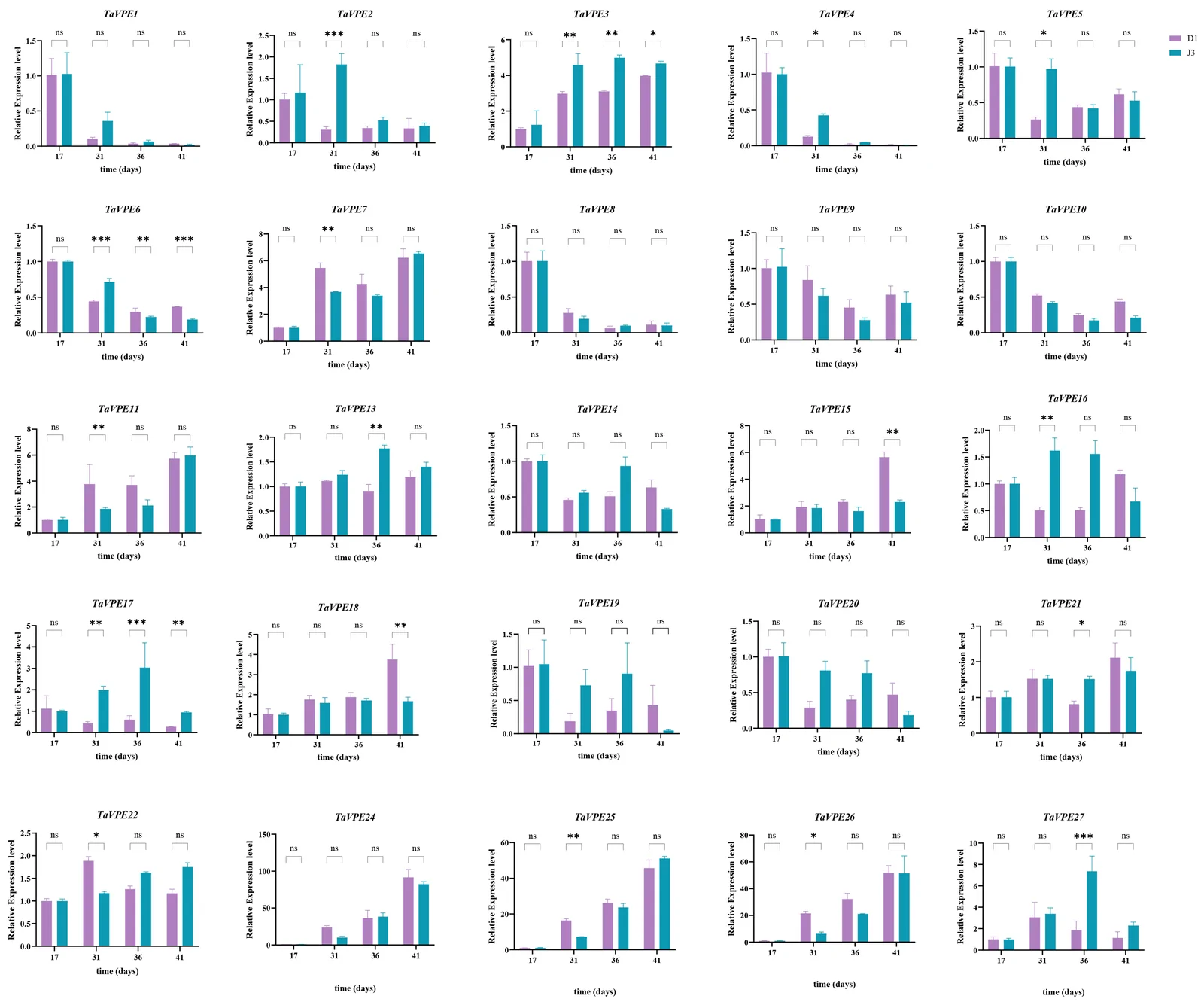
RT-qPCR analysis of *TaVPE* gene expression in the developing grains of D1 and J3. Relative expression levels of 25 *TaVPE* genes were determined in grains of the purple-grained cultivar Donghei-1 (D1, purple bars) and the white-grained cultivar Jinmai-66 (J3, teal bars) at 17, 31, 36 and 41 days after flowering (DAF). Gene expression was normalized to the wheat reference gene (*TaActin*) using the 2^−ΔΔCt^ method, and the expression level of D1 at 17 DAF was set to 1. Data are means ± SD of three biological replicates. Asterisks indicate significant differences between D1 and J3 at the same stage (Student’s *t*-test): * *p* < 0.05, ** *p* < 0.01, *** *p* < 0.001; ns, not significant (*p* ≥ 0.05).

To validate the transcriptome-based expression patterns, the relative expression levels of 25 *TaVPE* genes in developing grains of D1 (purple grain) and J3 (white grain) at 17, 31, 36 and 41 DAF were determined by RT-qPCR (Figure 10, Table S8). The overall expression trends were highly consistent with the transcriptome data: *TaVPE24*, *TaVPE25* and *TaVPE26* were barely expressed at 17 DAF but were strongly induced during grain filling, with their relative expression levels at 41 DAF being dozens of times higher than at 17 DAF, whereas *TaVPE1*, *TaVPE8*, *TaVPE9*, *TaVPE10* and *TaVPE14* maintained low and stable expression across the four stages.

Significant genotype-dependent differences between D1 and J3 were detected for 17 *TaVPE* genes at one or more stages. At 31 DAF, *TaVPE7*, *TaVPE11*, *TaVPE22*, *TaVPE25* and *TaVPE26* were expressed at significantly higher levels in D1 than in J3 (*p* < 0.05), whereas *TaVPE2*, *TaVPE3*, *TaVPE4*, *TaVPE5*, *TaVPE6*, *TaVPE16* and *TaVPE17* were significantly higher in J3. At 36 DAF, only *TaVPE6* was significantly more highly expressed in D1, while *TaVPE3*, *TaVPE13*, *TaVPE17*, *TaVPE21* and *TaVPE27* were significantly higher in J3. At 41 DAF, *TaVPE6*, *TaVPE15* and *TaVPE18* were significantly higher in D1, whereas *TaVPE3* and *TaVPE17* remained significantly higher in J3. Notably, *TaVPE3* and *TaVPE17* were significantly more highly expressed in J3 at three consecutive stages (31, 36 and 41 DAF), while *TaVPE6* showed a stage-dependent switch: significantly higher in J3 at 31 DAF but significantly higher in D1 at 36 and 41 DAF. Although *TaVPE24* expression in D1 was consistently higher than in J3 from 31 to 41 DAF, the difference did not reach statistical significance. The remaining genes (*TaVPE1*, *TaVPE8*, *TaVPE9*, *TaVPE10*, *TaVPE14*, *TaVPE19* and *TaVPE20*) showed no significant difference between the two cultivars at any stage. Taken together, a large proportion of TaVPE members are differentially expressed between purple- and white-grained wheat during grain development, the D1-biased expression of *TaVPE6*, *TaVPE15*, *TaVPE18*, *TaVPE25* and *TaVPE26* during the middle and late grain-filling stages identifies these five genes as candidates associated with purple grain colour.

## 4. Discussion

The 27 *TaVPE* genes identified in this study substantially outnumber the *VPE* members in *Arabidopsis* (4) and rice (5), which is primarily attributable to the allohexaploid nature of bread wheat [3,5]. Most *TaVPE* members occurred as triplets with a clear one-to-one homoeologous correspondence among the A, B and D subgenomes, indicating that whole-genome duplication (polyploidization) was the principal driving force for family expansion. In addition, the tandem gene clusters on homoeologous group 2 chromosomes (*TaVPE1*-*TaVPE11*) reflect local expansion. All duplicated *TaVPE* pairs exhibited Ka/Ks ratios below 1 (0.060–0.466), indicating that the *TaVPE* family has evolved under purifying selection, which acts to eliminate deleterious mutations and preserve the essential proteolytic functions of *VPEs* [49]. The breadth of this range is itself informative: the relatively higher ratio of *TaVPE5*/*TaVPE9* (0.466), although still indicative of purifying selection, points to weaker functional constraint in this pair and is consistent with the structural deviations observed for *TaVPE5* and *TaVPE6* (shortened N-terminus and loss of several conserved motifs), suggesting some relaxation of constraint or sub-functionalization after duplication. These interpretations are inferred from sequence data and require experimental confirmation.

Structurally, all 27 TaVPE proteins retained the two hallmark domains of the legumain family, namely the Peptidase_C13 catalytic domain and the C-terminal legumain_C domain [27,43], and motif composition and gene structure were highly conserved within each phylogenetic group, supporting the reliability of the classification into four groups. The observed structural deviations, such as the loss of several motifs and the shortened N-terminus of TaVPE5 and TaVPE6, indicate that these two members are more divergent than the rest of the family. The predominantly acidic isoelectric points of most TaVPE proteins are consistent with the acidic vacuolar environment in which VPE zymogens are activated and function [27,29]. Their assignment to the extracellular/secretory category by WoLF PSORT should be interpreted as evidence of signal-peptide-dependent routing through the secretory pathway rather than as their final localization, since the mature, active enzyme is expected to reside in the vacuole; definitive localization will require experimental validation.

Interspecific synteny analysis revealed 47 orthologous VPE pairs between wheat and the three monocots rice, maize and foxtail millet, with a strict one-to-two correspondence between each syntenic *TaVPE* gene and two orthologous loci in each monocot genome. The absence of collinear VPE pairs between wheat and *Arabidopsis* is consistent with the deep evolutionary divergence between monocots and dicots and with the extensive genome rearrangements that followed their separation [48]; lineage-specific gene loss in *Arabidopsis* and differences in genome assembly completeness provide alternative explanations. The retention of VPE-anchored syntenic blocks across rice, maize and foxtail millet nevertheless supports the interpretation that these loci predate the radiation of the Poaceae. These syntenic loci therefore originated from ancient genome duplication events and have been stably maintained during monocot evolution, which is consistent with the essential and conserved roles of *VPEs* in cereal seed maturation, such as glutelin processing in rice [32,33] and the ABA-coupled maturation and PCD programmes in barley [46]. In contrast, no collinear *VPE* pair was detected between wheat and *Arabidopsis*, reflecting the deep evolutionary divergence between monocots and dicots and the extensive genome rearrangements that followed their separation [48].

Promoter analysis showed that ABA-responsive elements (ABREs) were present in all 27 *TaVPE* promoters and constituted the most abundant *cis*-acting element category, followed by MeJA- and SA-responsive elements. ABA is a central hormone governing grain filling, maturation and desiccation tolerance [46], and it also positively regulates anthocyanin biosynthesis in fruits and reproductive tissues [16,17,74,75,76]; the universal presence of ABREs therefore suggests that *TaVPE* expression is integrated into ABA-mediated grain maturation and pigmentation programmes. The enrichment of jasmonate- and salicylic acid-responsive elements is consistent with the established roles of VPEs in defense-related cell death [37,38,39,40,41]. Notably, a large number of MYB- and bHLH/MYC-binding sites were detected in *TaVPE* promoters, implying potential crosstalk between *TaVPE* genes and the MBW transcriptional complex that controls anthocyanin biosynthesis in wheat pericarp [13,14,15,25,77,78].

Expression analysis based on public datasets and our own transcriptomes showed that *TaVPE* genes were preferentially expressed at anthesis and during grain filling, a developmental window that coincides with endosperm PCD [35,36,79,80], storage product accumulation and, in purple wheat, anthocyanin deposition in the pericarp [6,22,24,81,82,83]. In the comparative transcriptomes of the purple-grained cultivar D1 and the white-grained cultivar J3, transcriptional divergence increased as grain development progressed and became increasingly biased toward genes with higher expression in D1 at the late stages, consistent with recent multi-omics dissections of wheat grain filling [84] and comparative transcriptomics of pigmented cereal pericarps [85]. Among the *TaVPE* members, *TaVPE24* and *TaVPE25* (Group II homoeologues on group 6 chromosomes) showed the strongest D1-biased response in the RNA-seq comparison at 36 and 41 DAF (more than 15- and 3-fold, respectively), and *TaVPE4* showed markedly higher expression in D1 at 36 DAF; RT-qPCR confirmed significant D1-biased expression of *TaVPE25* and *TaVPE26* at 31 DAF and of *TaVPE6*, *TaVPE15* and *TaVPE18* at 36 and 41 DAF, and therefore identified *TaVPE6*, *TaVPE15*, *TaVPE18*, *TaVPE25* and *TaVPE26* as the five candidate genes. *TaVPE3* and *TaVPE17*, by contrast, were consistently more highly expressed in the white-grained J3 at three consecutive stages, indicating that different *TaVPE* members may show distinct, or even opposing, transcriptional behaviour during grain development and pigmentation. A plausible but as yet untested hypothesis for the association between *TaVPE* expression and purple grain colour is that VPE-mediated vacuolar processing could modulate the maturation of enzymes or transporters of the flavonoid pathway, such as anthocyanin-related glutathione S-transferases responsible for vacuolar sequestration [86,87], and that VPE-dependent PCD and tonoplast dynamics in pericarp cells may influence the deposition and stabilization of anthocyanins [28,29]. We emphasise that this model is a hypothesis generated by, rather than tested in, the present study: no VPE activity, GST activity, vacuolar transport or anthocyanin flux was measured here, and the role of GSTs is invoked by analogy with other systems rather than as a demonstrated interaction in wheat. To our knowledge, no study has yet directly demonstrated VPE involvement in anthocyanin accumulation or vacuolar sequestration in any pigmented tissue, and this absence of prior direct evidence is part of the rationale for the present, hypothesis-generating work.

To our knowledge, this study provides the first genome-wide characterization of the *VPE* gene family in wheat and the first transcriptomic evidence associating *VPE* members with grain pigmentation. Applying one uniform selection rule throughout, the same five genes (*TaVPE6*, *TaVPE15*, *TaVPE18*, *TaVPE25* and *TaVPE26*) are reported as candidate genes. These five genes constitute promising entry points for functional studies and for marker-assisted breeding of anthocyanin-rich wheat cultivars, which are of growing interest for the production of functional foods [6,7,8,10,11]. Several limitations of this study should be acknowledged. First, the comparison of grain colour was made between two cultivars that differ at many loci beyond the genes controlling pigmentation, so grain colour and genotype are fully confounded in this two-cultivar design; the observed expression differences may therefore reflect general developmental or background variation unrelated to anthocyanin biosynthesis. Second, anthocyanin content was not quantified in the present study, so the coincidence between *TaVPE* expression and pigment deposition could not be assessed directly. Third, the predicted subcellular localizations rest on sequence-based inference and require experimental confirmation. Finally, although *TaVPE24* showed a consistent D1-biased trend in RT-qPCR assays, the difference did not reach statistical significance; this may reflect differences in primer efficiency, alternative transcript isoforms, biological variation or sampling noise, because the qPCR primers may not target the same transcript isoform quantified by RNA-seq. *TaVPE24* was therefore excluded from the candidate set under the selection rule. Consequently, the data presented here establish an association at the transcript level and not a causal link. To disentangle pigment-associated regulation from cultivar background, future work should compare near-isogenic lines or segregating populations that differ only in grain colour, and should quantify anthocyanin content in the same material. Definitive roles of the candidate *TaVPE* genes will require functional experiments, including overexpression and CRISPR-Cas9 knockout, enzyme activity assays, experimental subcellular localization (e.g., fluorescence-tagged transient expression) and promoter interaction assays (e.g., yeast one-hybrid or dual-luciferase) to test the predicted MBW-mediated regulation. Such studies will clarify whether VPEs contribute to grain colour formation and will facilitate the exploitation of coloured wheat in breeding programmes.

## 5. Conclusions

We performed the first genome-wide analysis of the vacuolar processing enzyme (VPE) family in bread wheat (*Triticum aestivum* L.) and identified 27 *TaVPE* members clustered into four phylogenetic clades, all containing conserved Peptidase_C13 and legumain_C domains. Distributed unevenly across 12 chromosomes of four homoeologous groups, this family expanded through polyploidization and duplications, and its 26 collinear pairs experienced strong purifying selection. Interspecific synteny revealed 47 monocot ortholog pairs but no *Arabidopsis* counterparts, indicating Poaceae-conserved *VPE* loci. Abundant hormone-responsive and MYB/bHLH-binding cis-acting elements in the promoters suggest connections with grain maturation and anthocyanin biosynthesis. Using public datasets and transcriptomes of purple-grained Donghei-1 and white-grained Jinmai-66 across four grain-filling stages, we found *TaVPE* genes predominantly expressed during anthesis and grain filling. Under one uniform selection rule, five genes (*TaVPE6*, *TaVPE15*, *TaVPE18*, *TaVPE25* and *TaVPE26*) were identified as candidates associated with purple grain colour, each showing significant purple-biased expression that was confirmed by RT-qPCR. This study therefore provides a wheat *VPE* family resource and a set of candidate genes for future functional research and breeding. Further experiments including anthocyanin quantification, comparison of near-isogenic lines, overexpression, CRISPR-Cas9 knockout, enzyme assays and MBW-complex crosstalk analysis are required to validate the candidates.

## Figures and Tables

**Table 1:** Physicochemical properties and predicted subcellular localization of TaVPE proteins.

Gene Name	Gene ID	AA (aa)	MW (kDa)	pI	Instability Index	Aliphatic Index	GRAVY	Subcellular Localization Prediction
*TaVPE1*	*TraesCS2A02G385900*	515	56.96	6.11	42.94	76.33	−0.340	Extracellular
*TaVPE2*	*TraesCS2A02G386600*	491	54.23	6.29	37.65	70.53	−0.437	Extracellular
*TaVPE3*	*TraesCS2A02G386700*	492	54.11	6.22	38.47	74.59	−0.343	Extracellular
*TaVPE4*	*TraesCS2B02G402800*	495	54.76	5.91	44.40	75.68	−0.389	Extracellular
*TaVPE5*	*TraesCS2B02G404000*	361	40.43	6.72	40.49	84.52	−0.239	Mitochondria, Cytosol
*TaVPE6*	*TraesCS2B02G404200*	395	44.16	6.08	40.57	66.63	−0.544	Cytosol
*TaVPE7*	*TraesCS2B02G404300*	492	54.43	6.65	40.61	74.59	−0.390	Cytosol
*TaVPE8*	*TraesCS2D02G382400*	499	55.02	6.74	44.62	72.16	−0.397	Extracellular
*TaVPE9*	*TraesCS2D02G383300*	449	49.57	7.96	37.65	76.73	−0.307	Extracellular
*TaVPE10*	*TraesCS2D02G383400*	494	54.55	6.33	39.11	70.51	−0.432	Extracellular, Cytosol
*TaVPE11*	*TraesCS2D02G383500*	488	53.86	6.48	37.16	74.00	−0.374	Extracellular, Cytosol
*TaVPE12*	*TraesCS3A02G204500*	493	53.86	5.84	42.69	77.40	−0.294	Extracellular
*TaVPE13*	*TraesCS3A02G528800*	475	52.28	5.58	43.27	79.89	−0.265	Extracellular
*TaVPE14*	*TraesCS3A02G528900*	475	52.17	5.75	41.26	81.73	−0.263	Extracellular
*TaVPE15*	*TraesCS3B02G233000*	493	54.01	5.84	41.95	78.17	−0.276	Extracellular
*TaVPE16*	*TraesCS3B02G597800*	476	52.21	5.64	43.52	81.37	−0.237	Extracellular
*TaVPE17*	*TraesCS3B02G597900*	490	53.59	6.01	38.46	81.61	−0.285	Extracellular
*TaVPE18*	*TraesCS3D02G207600*	493	53.93	5.76	42.60	76.41	−0.286	Extracellular
*TaVPE19*	*TraesCS3D02G534100*	475	52.19	5.58	44.84	78.25	−0.265	Extracellular
*TaVPE20*	*TraesCS3D02G534200*	488	53.34	5.92	39.76	80.55	−0.287	Extracellular
*TaVPE21*	*TraesCS5A02G228900*	473	51.08	5.24	45.75	80.87	−0.284	Extracellular
*TaVPE22*	*TraesCS5B02G227600*	473	51.24	5.37	43.71	77.99	−0.326	Extracellular
*TaVPE23*	*TraesCS5D02G238800*	474	51.34	5.62	44.97	78.86	−0.329	Extracellular
*TaVPE24*	*TraesCS6A02G238500*	492	54.66	5.81	43.92	76.75	−0.377	Extracellular
*TaVPE25*	*TraesCS6B02G285900*	487	54.02	5.64	43.08	76.74	−0.369	Extracellular
*TaVPE26*	*TraesCS6D02G220900*	489	53.97	5.53	41.56	75.83	−0.359	Extracellular
*TaVPE27*	*TraesCS6D02G344700*	434	47.53	5.14	40.39	79.79	−0.291	Extracellular, Cytosol

Abbreviations: AA, amino acid length; MW, molecular weight (kDa); pI, theoretical isoelectric point; GRAVY, grand average of hydropathicity.

## Data Availability

The original contributions presented in this study are included in the article. Further inquiries can be directed to the first author.

## Supplementary Materials

The following supporting information can be downloaded at: https://www.mdpi.com/article/10.3390/biology15201800/s1, Table S1: Identification and physicochemical properties of the *TaVPE* genes in wheat (*Triticum aestivum* L.); Table S2: Gene identifiers and nomenclature of the *TaVPE* genes, with the *Arabidopsis* (At) and rice (Os) *VPE* genes used as references; Table S3: Non-synonymous (Ka) and synonymous (Ks) substitution rates and Ka/Ks ratios of paralogous *TaVPE* gene pairs; Table S4: Orthologous relationships of the *TaVPE* genes with their homologues in rice (*Oryza sativa*), maize (*Zea mays*) and foxtail millet (*Setaria italica*); Table S5: Numbers of *cis*-acting regulatory elements predicted in the promoters of the *TaVPE* genes; Table S6: Expression profiles of the *TaVPE* genes across 12 developmental stages and four hormone treatments (public WheatOmics atlas); Table S7: FPKM values of the *TaVPE* genes in developing grains of the purple-grained line Donghei 1 (D1) and the white control Jinmai 66 (J3) at 17, 31, 36 and 41 days after flowering; Table S8: Primer sequences used for quantitative real-time PCR of the *TaVPE* genes.

## Abbreviations

The following abbreviations are used in this manuscript:

RT-qPCR	Reverse Transcription Quantitative Polymerase Chain Reaction
GRAVY	Grand Average of Hydropathy
pI	The Theoretical Isoelectric Point
ABA	abscisic acid
bHLH	Basic helix–loop–helix
FPKM	Fragments Per Kilobase of transcript per Million mapped reads
LTR	Low-Temperature-Responsive
PCA	Principal component analysis
TPM	Transcripts per million

## References

[1] Brenchley R., Spannagl M., Pfeifer M., Barker G.L.A., D’Amore R., Allen A.M., McKenzie N., Kramer M., Kerhornou A., Bolser D. (2012). Analysis of the bread wheat genome using whole-genome shotgun sequencing. Nature.

[2] Marcussen T., Sandve S.R., Heier L., Spannagl M., Pfeifer M., Jakobsen K.S., Wulff B.B.H., Steuernagel B., Mayer K.F.X., The International Wheat Genome Sequencing Consortium (2014). Ancient hybridizations among the ancestral genomes of bread wheat. Science.

[3] Appels R., Eversole K., Stein N., Feuillet C., Keller B., Rogers J., Pozniak C.J., Choulet F., Distelfeld A., The International Wheat Genome Sequencing Consortium (IWGSC) (2018). Shifting the limits in wheat research and breeding using a fully annotated reference genome. Science.

[4] Walkowiak S., Gao L., Monat C., Haberer G., Kassa M.T., Brinton J., Ramirez-Gonzalez R.H., Kolodziej M.C., Delorean E., Thambugala D. (2020). Multiple wheat genomes reveal global variation in modern breeding. Nature.

[5] Matsuoka Y. (2011). Evolution of Polyploid *Triticum* Wheats under Cultivation: The Role of Domestication, Natural Hybridization and Allopolyploid Speciation in their Diversification. Plant Cell Physiol..

[6] Abdel-Aal E.S.M., Young J.C., Rabalski I. (2006). Anthocyanin Composition in Black, Blue, Pink, Purple, and Red Cereal Grains. J. Agric. Food Chem..

[7] He J., Giusti M.M. (2010). Anthocyanins: Natural Colorants with Health-Promoting Properties. Annu. Rev. Food Sci. Technol..

[8] Garg M., Chawla M., Chunduri V., Kumar R., Sharma S., Sharma N.K., Kaur N., Kumar A., Mundey J.K., Saini M.K. (2016). Transfer of grain colors to elite wheat cultivars and their characterization. J. Cereal Sci..

[9] Li X., Wang F., Ta N., Huang J. (2025). The compositions, characteristics, health benefits and applications of anthocyanins in *Brassica* crops. Front. Plant Sci..

[10] Sharma N., Kumari A., Chunduri V., Kaur S., Banda J., Goyal A., Garg M. (2022). Anthocyanin biofortified black, blue and purple wheat exhibited lower amino acid cooking losses than white wheat. LWT.

[11] Francavilla A., Joye I.J. (2022). Anthocyanin Content of Crackers and Bread Made with Purple and Blue Wheat Varieties. Molecules.

[12] Winkel-Shirley B. (2001). Flavonoid Biosynthesis. A Colorful Model for Genetics, Biochemistry, Cell Biology, and Biotechnology. Plant Physiol..

[13] Ramsay N.A., Glover B.J. (2005). MYB–bHLH–WD40 protein complex and the evolution of cellular diversity. Trends Plant Sci..

[14] Gonzalez A., Zhao M., Leavitt J.M., Lloyd A.M. (2008). Regulation of the anthocyanin biosynthetic pathway by the TTG1/bHLH/Myb transcriptional complex in *Arabidopsis* seedlings. Plant J..

[15] Xu W., Dubos C., Lepiniec L. (2015). Transcriptional control of flavonoid biosynthesis by MYB–bHLH–WDR complexes. Trends Plant Sci..

[16] Jaakola L. (2013). New insights into the regulation of anthocyanin biosynthesis in fruits. Trends Plant Sci..

[17] Petroni K., Tonelli C. (2011). Recent advances on the regulation of anthocyanin synthesis in reproductive organs. Plant Sci..

[18] Himi E., Noda K. (2005). Red grain colour gene (*R*) of wheat is a Myb-type transcription factor. Euphytica.

[19] Khlestkina E.K., Röder M.S., Börner A. (2010). Mapping genes controlling anthocyanin pigmentation on the glume and pericarp in tetraploid wheat (*Triticum durum* L.). Euphytica.

[20] Efremova T.T., Morozov S.V., Chernyak E.I., Chumanova E.V. (2023). Combining the genes of blue aleurone and purple pericarp in the genotype of spring bread wheat Saratovskaya 29 to increase anthocyanins in grain. J. Cereal Sci..

[21] Glagoleva A., Kukoeva T., Mursalimov S., Khlestkina E., Shoeva O. (2022). Effects of Combining the Genes Controlling Anthocyanin and Melanin Synthesis in the Barley Grain on Pigment Accumulation and Plant Development. Agronomy.

[22] Shoeva O., Gordeeva E., Khlestkina E. (2014). The Regulation of Anthocyanin Synthesis in the Wheat Pericarp. Molecules.

[23] Zhang S., Sun F., Zhang C., Zhang M., Wang W., Zhang C., Xi Y. (2022). Anthocyanin Biosynthesis and a Regulatory Network of Different-Colored Wheat Grains Revealed by Multiomics Analysis. J. Agric. Food Chem..

[24] Tereshchenko O.Y., Arbuzova V.S., Khlestkina E.K. (2013). Allelic state of the genes conferring purple pigmentation in different wheat organs predetermines transcriptional activity of the anthocyanin biosynthesis structural genes. J. Cereal Sci..

[25] Jiang W., Liu T., Nan W., Jeewani D.C., Niu Y., Li C., Wang Y., Shi X., Wang C., Wang J. (2018). Two transcription factors *TaPpm1* and *TaPpb1* co-regulate anthocyanin biosynthesis in purple pericarps of wheat. J. Exp. Bot..

[26] Li J., Zhang C., Xu X., Su Y., Gao Y., Yang J., Xie C., Ma J. (2024). A MYB family transcription factor *TdRCA1* from wild emmer wheat regulates anthocyanin biosynthesis in coleoptile. Theor. Appl. Genet..

[27] Dall E., Brandstetter H. (2013). Mechanistic and structural studies on legumain explain its zymogenicity, distinct activation pathways, and regulation. Proc. Natl. Acad. Sci. USA.

[28] Hara-Nishimura I., Hatsugai N., Nakaune S., Kuroyanagi M., Nishimura M. (2005). Vacuolar processing enzyme: An executor of plant cell death. Curr. Opin. Plant Biol..

[29] Rojo E., Zouhar J., Carter C., Kovaleva V., Raikhel N.V. (2003). A unique mechanism for protein processing and degradation in *Arabidopsis thaliana*. Proc. Natl. Acad. Sci. USA.

[30] Wleklik K., Borek S. (2023). Vacuolar Processing Enzymes in Plant Programmed Cell Death and Autophagy. Int. J. Mol. Sci..

[31] Gruis D.F., Selinger D.A., Curran J.M., Jung R. (2002). Redundant Proteolytic Mechanisms Process Seed Storage Proteins in the Absence of Seed-Type Members of the Vacuolar Processing Enzyme Family of Cysteine Proteases. Plant Cell.

[32] Wang Y., Zhu S., Liu S., Jiang L., Chen L., Ren Y., Han X., Liu F., Ji S., Liu X. (2009). The vacuolar processing enzyme *OsVPE1* is required for efficient glutelin processing in rice. Plant J..

[33] Kumamaru T., Uemura Y., Inoue Y., Takemoto Y., Uddin Siddiqui S., Ogawa M., Hara-Nishimura I., Satoh H. (2010). Vacuolar Processing Enzyme plays an Essential Role in the Crystalline Structure of Glutelin in Rice Seed. Plant Cell Physiol..

[34] Nakaune S., Yamada K., Kondo M., Kato T., Tabata S., Nishimura M., Hara-Nishimura I. (2005). A Vacuolar Processing Enzyme, *δVPE*, Is Involved in Seed Coat Formation at the Early Stage of Seed Development. Plant Cell.

[35] Young T.E., Gallie D.R. (2000). Programmed cell death during endosperm development. Plant Mol. Biol..

[36] Sabelli P.A., Larkins B.A. (2009). The Development of Endosperm in Grasses. Plant Physiol..

[37] Hatsugai N., Kuroyanagi M., Yamada K., Meshi T., Tsuda S., Kondo M., Nishimura M., Hara-Nishimura I. (2004). A Plant Vacuolar Protease, VPE, Mediates Virus-Induced Hypersensitive Cell Death. Science.

[38] Kuroyanagi M., Yamada K., Hatsugai N., Kondo M., Nishimura M., Hara-Nishimura I. (2005). Vacuolar Processing Enzyme Is Essential for Mycotoxin-induced Cell Death in *Arabidopsis thaliana*. J. Biol. Chem..

[39] Rojo E., Martín R., Carter C., Zouhar J., Pan S., Plotnikova J., Jin H., Paneque M., Sánchez-Serrano J.J., Baker B. (2004). *VPEγ* Exhibits a Caspase-like Activity that Contributes to Defense against Pathogens. Curr. Biol..

[40] Misas-Villamil J.C., Toenges G., Kolodziejek I., Sadaghiani A.M., Kaschani F., Colby T., Bogyo M., van der Hoorn R.A.L. (2013). Activity profiling of vacuolar processing enzymes reveals a role for VPE during oomycete infection. Plant J..

[41] Misas-Villamil J.C., van der Hoorn R.A.L., Doehlemann G. (2016). Papain-like cysteine proteases as hubs in plant immunity. New Phytol..

[42] Gao X.X., Tang Y.L., Shi Q.Y., Wei Y.S., Wang X.X., Shan W.X., Qiang X.Y. (2023). Vacuolar processing enzyme positively modulates plant resistance and cell death in response to *Phytophthora parasitica* infection. J. Integr. Agric..

[43] van der Hoorn R.A.L. (2008). Plant Proteases: From Phenotypes to Molecular Mechanisms. Annu. Rev. Plant Biol..

[44] Kinoshita T., Yamada K., Hiraiwa N., Kondo M., Nishimura M., Hara-Nishimura I. (1999). Vacuolar processing enzyme is up-regulated in the lytic vacuoles of vegetative tissues during senescence and under various stressed conditions. Plant J..

[45] Deng H., Cao S., Zhang G., Xiao Y., Liu X., Wang F., Tang W., Lu X. (2024). *OsVPE2*, a Member of Vacuolar Processing Enzyme Family, Decreases Chilling Tolerance of Rice. Rice.

[46] Sreenivasulu N., Radchuk V., Strickert M., Miersch O., Weschke W., Wobus U. (2006). Gene expression patterns reveal tissue-specific signaling networks controlling programmed cell death and ABA-regulated maturation in developing barley seeds. Plant J..

[47] Lynch M., Conery J.S. (2000). The Evolutionary Fate and Consequences of Duplicate Genes. Science.

[48] Panchy N., Lehti-Shiu M., Shiu S.H. (2016). Evolution of Gene Duplication in Plants. Plant Physiol..

[49] Hurst L.D. (2002). The Ka/Ks ratio: Diagnosing the form of sequence evolution. Trends Genet..

[50] Zhang X., Yu Y., Sun Y., Bai Y., Shu Y., Guo C. (2024). Genome-Wide Identification and Expression Analysis of SNAP Gene Family in Wheat. Genes.

[51] Duan X., Zhang K., Yu Y., Liu Z. (2024). Genome-Wide Analysis of Soybean Vacuolar Processing Enzyme Gene Family Reveals Their Roles in Plant Development and Response to Stress Conditions. J. Plant Growth Regul..

[52] Finn R.D., Clements J., Eddy S.R. (2011). HMMER web server: Interactive sequence similarity searching. Nucleic Acids Res..

[53] Camacho C., Coulouris G., Avagyan V., Ma N., Papadopoulos J., Bealer K., Madden T.L. (2009). BLAST+: Architecture and applications. BMC Bioinform..

[54] Wang J., Chitsaz F., Derbyshire M.K., Gonzales N.R., Gwadz M., Lu S., Marchler G.H., Song J.S., Thanki N., Yamashita R.A. (2023). The conserved domain database in 2023. Nucleic Acids Res..

[55] Mistry J., Chuguransky S., Williams L., Qureshi M., Salazar G.A., Sonnhammer E.L.L., Tosatto S.C.E., Paladin L., Raj S., Richardson L.J. (2021). Pfam: The protein families database in 2021. Nucleic Acids Res..

[56] Gasteiger E., Hoogland C., Gattiker A., Duvaud S.E., Wilkins M.R., Appel R.D., Bairoch A., Walker J.M. (2005). Protein Identification and Analysis Tools on the ExPASy Server. The Proteomics Protocols Handbook.

[57] Kyte J., Doolittle R.F. (1982). A simple method for displaying the hydropathic character of a protein. J. Mol. Biol..

[58] Horton P., Park K.J., Obayashi T., Fujita N., Harada H., Adams-Collier C.J., Nakai K. (2007). WoLF PSORT: Protein localization predictor. Nucleic Acids Res..

[59] Tamura K., Stecher G., Kumar S. (2021). MEGA11: Molecular Evolutionary Genetics Analysis Version 11. Mol. Biol. Evol..

[60] Letunic I., Bork P. (2024). Interactive Tree of Life (iTOL) v6: Recent updates to the phylogenetic tree display and annotation tool. Nucleic Acids Res..

[61] Bailey T.L., Boden M., Buske F.A., Frith M., Grant C.E., Clementi L., Ren J., Li W.W., Noble W.S. (2009). MEME SUITE: Tools for motif discovery and searching. Nucleic Acids Res..

[62] Hu B., Jin J., Guo A.Y., Zhang H., Luo J., Gao G. (2015). GSDS 2.0: An upgraded gene feature visualization server. Bioinformatics.

[63] Chen C., Wu Y., Li J., Wang X., Zeng Z., Xu J., Liu Y., Feng J., Chen H., He Y. (2023). TBtools-II: A “one for all, all for one” bioinformatics platform for biological big-data mining. Mol. Plant.

[64] Wang Y., Tang H., DeBarry J.D., Tan X., Li J., Wang X., Lee T.H., Jin H., Marler B., Guo H. (2012). MCScanX: A toolkit for detection and evolutionary analysis of gene synteny and collinearity. Nucleic Acids Res..

[65] Zhang Z., Li J., Zhao X.Q., Wang J., Wong G.K.S., Yu J. (2006). KaKs_Calculator: Calculating Ka and Ks Through Model Selection and Model Averaging. Genom. Proteom. Bioinform..

[66] Lescot M. (2002). PlantCARE, a database of plant *cis*-acting regulatory elements and a portal to tools for in silico analysis of promoter sequences. Nucleic Acids Res..

[67] Ma S., Wang M., Wu J., Guo W., Chen Y., Li G., Wang Y., Shi W., Xia G., Fu D. (2021). WheatOmics: A platform combining multiple omics data to accelerate functional genomics studies in wheat. Mol. Plant.

[68] Bolger A.M., Lohse M., Usadel B. (2014). Trimmomatic: A flexible trimmer for Illumina sequence data. Bioinformatics.

[69] Dobin A., Davis C.A., Schlesinger F., Drenkow J., Zaleski C., Jha S., Batut P., Chaisson M., Gingeras T.R. (2013). STAR: Ultrafast universal RNA-seq aligner. Bioinformatics.

[70] Danecek P., Bonfield J.K., Liddle J., Marshall J., Ohan V., Pollard M.O., Whitwham A., Keane T., McCarthy S.A., Davies R.M. (2021). Twelve years of SAMtools and BCFtools. GigaScience.

[71] Li B., Dewey C.N. (2011). RSEM: Accurate transcript quantification from RNA-Seq data with or without a reference genome. BMC Bioinform..

[72] Love M.I., Huber W., Anders S. (2014). Moderated estimation of fold change and dispersion for RNA-seq data with DESeq2. Genome Biol..

[73] Livak K.J., Schmittgen T.D. (2001). Analysis of Relative Gene Expression Data Using Real-Time Quantitative PCR and the 2^−ΔΔCT^ Method. Methods.

[74] Arzumanova K., Rohlfs R.V., Grønvold L., Strand M.A., Hvidsten T.R., Sandve S.R. (2023). Analyses of Genome Regulatory Evolution Following Whole-Genome Duplication Using the Phylogenetic EVE Model. Methods in Molecular Biology.

[75] Ji X., Li Z., Zhang M., Lang S., Song X. (2024). *ChMYB1*-*ChbHLH42*-*ChTTG1* module regulates abscisic acid-induced anthocyanin biosynthesis in *Cerasus humilis*. Hortic. Plant J..

[76] Ma B., Song Y., Feng X., Guo Q., Zhou L., Zhang X., Zhang C. (2024). Exogenous Abscisic Acid Regulates Anthocyanin Biosynthesis and Gene Expression in Blueberry Leaves. Horticulturae.

[77] Sun X., Zhang Z., Li J., Zhang H., Peng Y., Li Z. (2022). Uncovering Hierarchical Regulation among MYB-bHLH-WD40 Proteins and Manipulating Anthocyanin Pigmentation in Rice. Int. J. Mol. Sci..

[78] Chen L., Cui Y., Yao Y., An L., Bai Y., Li X., Yao X., Wu K. (2023). Genome-wide identification of WD40 transcription factors and their regulation of the MYB-bHLH-WD40 (MBW) complex related to anthocyanin synthesis in Qingke (*Hordeum vulgare L.* var. *nudum* Hook. f.). BMC Genom..

[79] Pitsili E., Nowack M.K. (2026). Programmed cell death during plant development. Development.

[80] Hertig C., Rutten T., Melzer M., Schippers J.H.M., Thiel J. (2023). Dissection of Developmental Programs and Regulatory Modules Directing Endosperm Transfer Cell and Aleurone Identity in the Syncytial Endosperm of Barley. Plants.

[81] Xia Q., Shui Y., Zhi H., Ali A., Yang Z., Gao Z. (2023). Exogeneous selenium enhances anthocyanin synthesis during grain development of colored-grain wheat. Plant Physiol. Biochem..

[82] Thapa M., Liu L., Barkla B.J., Kretzschmar T., Rogiers S.Y., Rose T.J. (2024). Accumulation patterns of anthocyanin and γ-oryzanol during black rice grain development. PLoS ONE.

[83] Zhang M., Duan S., Zhang J., Fei R., Zhao X., Ji C., Liu L. (2026). Temporal Partitioning of Carotenoid, Flavonoid and Anthocyanin Biosynthesis Underlies the Ontogenetic Petal Color Transition in *Weigela japonica*. Metabolites.

[84] Wang X., Chen C., Zuo Y., Jiang G., Li H., Xie Y., Zhou J., Xia H., Zhang C., Qin P. (2026). Integrative multi-omics analysis uncovers the regulatory network of wheat grain development during grain filling. BMC Plant Biol..

[85] Holly A.A., Michael P.N., John J.A. (2026). Comparative transcriptomics of anthocyanin accumulation in the pericarp of pigmented purple corn. Theor. Appl. Genet..

[86] Fu M., Li G., Lu M., Jiang S., Karamat U., Khan I., Guo J. (2025). *BrGSTF12*, an anthocyanin-related glutathione S-transferase gene, is essential for light-induced anthocyanin accumulation in zicaitai (*Brassica Rapa Var. purpuraria*). BMC Plant Biol..

[87] Qin J., Zhao C., Wang S., Gao N., Wang X., Na X., Wang X., Bi Y. (2022). PIF4-PAP1 interaction affects MYB-bHLH-WD40 complex formation and anthocyanin accumulation in *Arabidopsis*. J. Plant Physiol..

